# Supplementary material for: Targeted SPP1 Inhibition of Tumor‐Associated Myeloid Cells Effectively Decreases Tumor Sizes
Source: Adv Sci (Weinh). 2024 Dec 5;12(4):2410360. doi: 10.1002/advs.202410360 (PMC11775521; doi:10.1002/advs.202410360)
Supplement: Supplementary file 1 — Supporting Information [file ADVS-12-2410360-s001.docx]

**Targeted SPP1 inhibition of tumor-associated myeloid cells effectively decreases tumor sizes**

Benan Kartal^1^, Christopher S. Garris^1^, Hyung Shik Kim^1^, Rainer H. Kohler^1^, Jasmine Carrothers^1^, Elias A. Halabi^1^, Yoshiko Iwamoto^1^, Anne-Gaëlle Goubet^2,3^, Yuxuan Xie^2,3^, Pratyaksha Wirapati^2,3^, Mikaël J. Pittet^2,3,4^, Ralph Weissleder^1,5,^*

^1^ Center for Systems Biology, Massachusetts General Hospital, 185 Cambridge St, CPZN 5206, Boston, MA 02114

^2^ Department of Pathology and Immunology, University of Geneva, Geneva, Switzerland

^3^ AGORA Cancer Research Center, Swiss Cancer Center Leman, Lausanne, Switzerland

^4^ Ludwig Institute for Cancer Research, Lausanne, Switzerland

^5^ Department of Systems Biology, Harvard Medical School, 200 Longwood Ave, Boston, MA 02115

*R. Weissleder, MD, PhD

Center for Systems Biology

Massachusetts General Hospital

185 Cambridge St, CPZN 5206

Boston, MA, 02114

617-726-8226

[rweissleder@mgh.harvard.edu](mailto:weissleder@helix.mgh.harvard.edu)

Keywords: nanoparticles, cancer, macrophage, SPP1

## Abstract

SPP1^High^ tumor-associated macrophages (TAM) are abundant tumor myeloid cells that are immunosuppressive, pro-tumorigenic, and have a highly negative prognostic factor. Despite this, there is a lack of efficient TAM-specific therapeutics capable of reducing SPP1 expression. Here, we report on a phenotypic screen to identify small molecule SPP1 modulators in macrophages. We identified several hits and incorporated them into a TAM-avid systemic nanoformulation. We show that the lead compound (CANDI460) can down-regulate SPP1 in vitro and in vivo and lead to tumor remissions in different murine models. These findings are important as they offer a promising avenue for developing novel therapeutic strategies targeting TAM.

## Introduction

Tumor-associated macrophages (TAM) are often abundant in solid cancers, sometimes comprising up to half of the tumor cell mass. Generally, these cells are pro-tumorigenic, assisting in immune suppression, invasion, angiogenesis, epithelial-mesenchymal transition (EMT), cell proliferation, and therapy resistance. Recent advances in scRNA sequencing have shed light on the composition of these diverse cells, and different phenotypes have been described. For example, a recent study found that a high expression of secreted phosphoprotein 1 (SPP1) by TAM is a major contributor to patient clinical outcomes^[1]^. In contrast, TAM expression of M2 macrophage markers (e.g., defined by CD163 or MRC1) was not associated with different clinical outcomes in the same patients^[1]^, supporting the notion that M2 phenotypes define a possible extreme of polarized macrophages in vitro but may not necessarily reflect TAMs in vivo^[2]^. This, together with many other studies^[3‑9]^ has rekindled the interest in SPP1 as a biomarker of adverse prognosis and as a possible therapeutic target.

SPP1 in humans and Spp1 in mice encodes for a multifunctional phosphoglycoprotein (osteopontin, OPN) secreted by various tumor-associated cells, mostly TAM and tumor cells^[10]^. Three major OPN receptor-interacting receptors activate overlapping oncogenic signaling pathways, including hypoxia inducible factor 2a (HIF2a), alpha-v beta-3 integrin (avB3; MEKK1) and alpha-4 beta-1 integrin (IKK, AKT, Ras). SPP1/Spp1 thus has a plethora of functions (cytokine, chemokine and signal transduction) due to modular structural motifs that provide interaction surfaces for different integrins and the CD44 receptor. Due to multiple post-translational modifications and binding receptor heterogeneity, therapeutic targeting of SPP1 action is complex. Furthermore, while numerous studies have linked macrophage Spp1 expression with adverse clinical phenotypes ^[1,11]^], it is unclear whether SPP1 is a biomarker, or a direct target. Genetic deletion of Spp1 in macrophages could reduce tissue fibrosis in cardiovascular disease models by influencing TGFb1 signaling. TGFb is a potent driver of cancer immune suppression, potentially linking Spp1^+^ macrophages with the inhibition of adaptive immunity^[12]^. Different therapeutic strategies have been proposed to inhibit SPP1, including the use of monoclonal antibodies, siRNA, aptamers, and small molecule inhibitors^[13,14]^. Despite these proposed approaches, no strategy currently exists to effectively antagonize Spp1 in TAM. Consequently, we explored a strategy to effectively antagonize Spp1 in TAM by re-polarizing TAM away from Spp1 phenotypes.

Here, we first determined whether small molecules could be used to polarize macrophages toward a SPP1/Spp1^Low^ phenotype. While some putative small molecule inhibitors of Spp1 have been proposed^[13]^, their comparative effectiveness and TAM specificity is largely unknown. We thus designed a cell-based screen using primary bone marrow-derived macrophages from *Spp1^tdTomato^* reporter mice^[15]^. This allowed us to compare different inhibitors and their combinations in a phenotypic screen. We hypothesized that multidrug combinations could be synergistic and thus formulated the most promising hits into a single TAM-avid polymeric drug delivery system, referred to as cyclodextrin-adjuvant nanoconstruct for dual immunotherapy (CANDI). We present the design of this nanoformulation and how it can be used to influence the phenotype of Spp1^High^ TAM. The findings are important as they provide a promising avenue for the development of novel therapeutic strategies targeting tumor-promoting TAM.

## Results

***Induction of Spp1 expression in TAM***

TAM are abundant in many solid tumors and have generally been associated with negative outcomes^[16,17]^, therapeutic resistance^[18,19]^ and tumor invasion^[20]^. Yet, macrophages are plastic, and different phenotypes have been identified by single cell RNAseq^[2]^. Notably, a recent study^[1]^ identified TAM-associated SPP1 expression as a key negative predictor of patient clinical outcome in various cancer types (**Fig. 1A-B**). Analysis of published single cell RNAseq datasets^[1,21,22]^ confirms this finding in several large human cancer cohorts (**Fig. S1**).

*SPP1/Spp1* production in TAM can be regulated by several factors (**Fig. 1C**), including tumor necrosis factor (TNF), interleukin 6 (IL-6), interleukin 1b (IL-1b), lipopolysaccharide (LPS), colony-stimulating factor 2 (CSF2, GM-CSF)^[23]^, macrophage migration inhibitory factor (MIF)^[24]^ and hypoxia^[1,25]^ or inversely by interferon regulatory factor 8 (IRF8)^[26,27]^. In turn, *SPP1* expression leads to the production of osteopontin, a molecule with pleiotropic effects^[23]^, and globally promoting different hallmarks of cancer (**Fig. 1C**).

To investigate *Spp1* expression induced in TAM, initially we used *Spp1^tdTomato^* reporter mice^[15]^ in which MC38-*H2B^GFP^* tumor cells were implanted in dorsal skinfold window chambers. We found large numbers (41.8%) of *Spp1^tdTomato^*-positive macrophages (positive for F4/80 and CD11b) in these tumors, as illustrated on day 7 after tumor implantation (**Fig. 1D**). Next we studied the temporal kinetics of *Spp1* expression in macrophages in vitro by using bone marrow cells from *Spp1^tdTomato^* reporter mice, and which were differentiated into macrophages over 8 days using well-established protocols^[28]^. We found that *Spp1* expression levels increased threefold with cell differentiation, reaching a plateau at around day 6. At this point, all bone marrow-derived macrophages showed extensive *Spp1* expression (~0.2 AU/cell; p<0.01) (**Fig. 1E**). Taken together, these data indicate the usefulness of the *Spp1^tdTomato^* reporter system to study the emergence of *Spp1^tdTomato^*-positive macrophages both in vivo and in vitro, and thus potentially how the induction of *Spp1* expression could be therapeutically inhibited, as explored below.

***Cell-based screen identifies TAM Spp1 inhibitors***

After showing the temporal induction of *Spp1* in myeloid cells, we set up a phenotypic screen to test the effects of putative small molecule modulators of SPP1. For this screen, we prioritized the use of primary cells isolated from donor mice over immortalized cell lines to more accurately represent TAM as seen in vivo. Due to this constraint, we opted for a focused approach, testing a curated set of 26 compounds identified from the literature and known to be associated with Spp1 modulation. The selection was based on established pathway interactions with Spp1, such as hypoxia^[29]^, MIF^[24]^, and peroxisome proliferator-activated receptor gamma (PPARG)^[30]^, as well as potential direct inhibitors.

Specifically, bone marrow cells from *Spp1^tdTomato^* reporter mice were harvested, differentiated into macrophages for 5 days, incubated with different concentrations (L: low, 0.1 µM; M: medium, 0.5 µM; H: high, 1 µM) of inhibitors on day 1 for 24 hours, and analyzed for *Spp1^tdTomato^* signal on day 5 (**Fig 2A**). Despite the limited availability of primary cells, we successfully screened at least 10^4^ cells per condition in duplicate at three different doses for each compound. This targeted strategy allowed us to effectively identify promising Spp1 inhibitors with relevance to TAM biology. Of interest, most putative Spp1 modulators actually did not decrease or prevent Spp1 expression levels in this screen, whereas compounds that showed a dose-dependent Spp1 inhibition included TLR agonists (R848, p=0.0196), CSF1Ri (PLX3397, p=0.0117 and PLX5622, p=0.0134), and an indirect TNFa inhibitor (shikonin, p=0.0398) (**Fig 2B**). With all three compounds there was near complete Spp1 inhibition as a function of dose (**Fig. 2C**).

Next, we tested whether the compounds could have additive or synergistic effects. We performed similar screens but with single, dual, and triple combinations of small molecule drugs (**Fig. 2D)**. Our data show that dual (R848 and PLX3397: 85.8%) and triple (R848 and PLX3397 and shikonin: 91.4%) combinations were more potent in *Spp1* inhibition over single drugs (both double and triple combinations: p<0.05). To determine the therapeutic windows (ED50 over LC50) of these three compound classes, we performed toxicity experiments (**Fig. S2**). We show broad therapeutic windows in the low µM range for R848 and PLX3397, but not for shikonin. Based on this information, we next designed a TAM delivery system to bring the top two hits (R848 and PLX3397) to TAM in vivo, which we named CANDI460, as described below. We performed molecular modeling (**Fig. S4** and **S5**) to determine whether R848 and PLX3397 would fit into the CANDI cavity, which they do. Using Autodock Vina, we docked R848 and PLX3397 into the central cavity of cyclodextrin. We found that in the highest-scoring docking poses, R848 maintains four hydrogen bonding interactions with the alcohols in the cavity, and PLX3397 maintains two hydrogen bonding interactions.

***CANDI460 nanoformulation: characterization and impact on Spp1^High^ macrophages***

The dually loaded, TAM-avid CANDI nanoformulation was synthesized by cross-linking bis-succinyl cyclodextrin using EDC (1-ethyl-3-(3-dimethylaminopropyl) carbodiimide hydrochloride) chemistry. The resulting nanoparticles had an average particle size of ~16 nm (**Fig. 3A**) and were monodispersed (**Fig. 3B**). The CANDI was well internalized into SPP1+ bone marrow-derived macrophages and appeared in lysosomal cellular compartments (**Fig. S9B**). CANDI could be loaded with R848 and PLX3397 at ~ 0.08mg small molecule per mg CANDI. The resulting CANDI460 (R848 0.25mg, PLX 0.142mg, size) was then used for additional efficacy and cellular toxicity studies using bone marrow-derived macrophages. The results show efficient (**Fig. 3D, E**) and safe (**Fig. 3F**) drug effects up to 0.2 mg/mL, well below the in vivo dose.

To evaluate the effects of CANDI460 on macrophage phenotypes, we conducted bulk RNA sequencing on bone marrow-derived macrophages treated or not with the nanoformulation. As anticipated, CANDI460 treatment significantly down-regulated the expression of *Spp1* (**Fig. 3G**)(p<0.05). Notably, the treatment also resulted in decreased expression of the mannose receptor *Mrc1* (also known as CD206), which is linked to alternative activation of macrophages, and *Trem2*, a membrane protein associated with tumor promotion and considered a candidate therapeutic target^[2]^. Furthermore, CANDI460 treatment triggered the upregulation of several inflammatory genes, including *Cxcl9* and interleukin *Il12a*, which are key players in promoting adaptive antitumor immunity^[1,31]^ (**Fig. 3G** and **Fig S8**). These findings indicate that CANDI460 can induce multiple phenotypic changes in TAM, which may contribute to enhanced tumor control.

***Therapeutic antitumor efficacy***

To determine whether these changes translate into anti-tumor efficacy, we conducted tumor growth experiments in the MC38 mouse model (**Fig. 4A**). The results showed significant antitumor effects following systemic administration of CANDI460. On day 15 after treatment, mean tumor volumes in the CANDI460 group were significantly lower (82±155mm3) compared to the control group (740±344mm3; p<0.0001; n=24; **Fig. 4B**). Three of the 12 CANDI-460 treated mice showed partial response to their treatment. We hypothesize that the resistance may be due to adaptive immune resistance, which is commonly seen as an inhibitory mechanism for anti-tumor immunity. Up-regulation of factors such as PD-L1, IDO, and TDO could be causing the partial responses observed. We find that in a difficult-to-treat melanoma model, the CANDI460 response can be boosted by PD-1 blockade, suggesting that adaptive resistance occurs in the model system.

Flow cytometry showed increased CD8 T cell infiltration (p<0.001; n=4) and decreased pro-tumorigenic TAM (**Fig. S7**)(p<0.05; n=3). Enhanced CD8 T cell presence in tumors could be due to CANDI460-induced expression of Cxcl9, Ccl5, and IL-12 (**Fig 3G**). These are all known factors that can enhance T cell-mediated anti-tumor immunity as Cxcl9 and Ccl5 are chemoattractive to T cells, and IL-12 is a potent activator of CD8 T cell effector functions.

Furthermore, 60 days after tumor inoculation, 9 of 12 mice (75%) in the CANDI460 group were still alive, compared to 0 of 12 mice (0%) in the control group (p<0.001; n=24)(**Fig. 4C**). The mice still alive showed a lack of tumor burden, both macroscopically (**Fig. 4E**) and microscopically (**Fig. 4E, Fig. S6**). These results indicate that CANDI460 not only targets SPP1 expression in TAM, but can also potently control tumor progression in mice and promote anti-tumor immune response. SPP1 macrophage polarization has been associated with negative clinical outcomes in cancer. Re-polarizing TAM away from Spp1-associated phenotypes can re-activate anti-tumor immunity by fostering productive myeloid-T cell communication, enabling immune rejection of tumors. Furthermore, cured mice were immune to tumor re-challenge (**Fig. 4D**). Innate immune cells can guide adaptive immune responses by controlling antigen presentation. Phagocytic myeloid cells, such as macrophages and dendritic cells, are known as professional antigen-presenting cells. In cancer, immune evasion by tumors can result from a block in antigen presentation and thus blockage of T cell stimulation. Immune suppressive myeloid cells, such as SPP1 macrophages, prevent tumor T cell responses. Re-polarizing the tumor macrophages to T cell supporting phenotypes (secreting Ccl5, Cxcl9, and IL-12, **Fig. 3G**) promotes T cell stimulation and adaptive immune recognition of tumors. This was seen after CANDI460 treatment, and we believe that this T cell response persists in mediating immune memory against cancer cells upon re-challenge (**Fig 4D)**.

To corroborate these results, we performed antitumor efficacy studies in a second model (**Fig. S12**). Using the B16F10 melanoma model^[28]^, we again observed remarkable findings. In the CANDI460 group, 2 of 5 mice survived, whereas in the group treated with CANDI460+ anti-PD1, 4 of 7 mice were cured (p<0.01; n=22).

***Intravital imaging reveals drug action in the tumor microenvironment***

We initially used intravital imaging to determine the pharmacokinetics and dynamics of CANDI460 using serial imaging in the MC38-TagBFP2 window chamber model. We observed a vascular half-life of approximately 2 hours, similar to other CANDI formulations^[32]^. Cellular accumulation in the TME was most pronounced at 24 hours after intravenous administration. At this time, the material primarily localized to tumor macrophages as determined by MerTK-GFP imaging (**Fig. S11**) and corroborative flow cytometry (**Fig. S9**).

To assess the pharmacodynamic effects of CANDI460, we performed additional longitudinal tumor imaging in *Spp1^tdTomato^* reporter mice. Specifically, we performed serial microscopic examinations of the tumor microenvironment 8 days after tumor implantation, both before and after intravenous systemic administration of CANDI460 (**Fig. 5A**). As expected, baseline expression of *Spp1* was high pre-treatment. However, within 48 hours post-infusion of CANDI460, we observed a significant reduction of *Spp1* expression in TAM as evidenced by the number of *Spp1*-positive cells (**Fig. 5B**, 10-fold, p<0.0001) and the *Spp1* signal intensity per cell (**Fig. 5C**,1-fold, p=0.01). Importantly, these changes were accompanied by tumor shrinkage, which was quite pronounced (e.g. see time course in **Fig. S10**).

## Discussion

TAM are often the most abundant cell types in tumors, with many of them being pro-tumorigenic^[2]^. Recent scRNA sequencing studies have shed light on the composition of human myeloid subsets in cancers^[1]^ and other inflammatory diseases^[11]^. One of the overarching discoveries of these studies was i) the high SPP1 expression in undesirable macrophages and ii) the virtual exclusiveness of SPP1 with other biomarkers of favorable macrophage subtypes (e.g. CXCL9). SPP1/Spp1 is a secreted phosphorylated protein produced by different cell types. It is expressed at low levels in circulating monocytes, and can be induced during their differentiation into macrophages, particularly in some of those found in tumors and sites of inflammation. Our knowledge of SPP1 function and induction originates mostly from work in cell lines including THP-1, RAW and HL-60 cells^[23,30]^ but has recently been substantiated by large scale scRNA sequencing studies in human tissues^[1,11]^. One of these studies found stereotyped gene expression programs revealing seven conserved TAM states in which only two preferentially expressed SPP1 (with or without MTH1) and almost mutually exclusive with antitumorigenic programs (e.g. CXCL9)^[1]^. These unique and opposed expression profiles^[1]^, as well as the emerging knowledge of SPP1 downstream effects^[23]^, form the basis of the current therapeutic strategy design. While we focus on TAM in this report, we argue that the discovered compounds could have similar therapeutic value in inflammatory conditions with high Spp1 levels. For example, in a recent study^[11]^, SPP1+ macrophages were shown to promote atrial fibrillation in the heart through cross-talk with local immune and stromal cells. This could be amenable to CANDI460 treatment.

Spp1 has a diverse range of effector functions, mediated in part through integrin binding. This enables Spp1 to affect multiple cell types and their functions^[10,27]^. Such broad activity suggests potential therapeutic modulation^[13,33]^. Notably, mouse studies have provided revealing data, demonstrating that *Spp1* knock-out mice have lower TAM infiltration and tumor growth compared to wild-type counterparts^[34,35]^. Various therapeutic strategies for Spp1 inhibition have been explored, including the use of siRNA, shRNA, aptamers, antibodies, and small-molecule inhibitors^[13,33]^ but efficacy has generally been modest. Interestingly, in our screening efforts, we found that many of the previously identified small molecules (e.g., andrographolide^[36]^) were inefficient, prompting a broader comparative screen.

Unexpectedly, we identified that the TLR7/8 agnostic R848 (also named resiquimod) and the CSFR1 inhibitors PLX3397 (also named pexidartinib) and PLX5622 had the highest efficacy in silencing Spp1 expression. R848, an imidazoquinoline, stimulates the NFkB pathway through TLR7/8 MyD88-dependent signaling, though its link to Spp1 down-regulation has not been reported. PLX3397, a CSFR1 inhibitor, has been extensively studied for its role in reducing TAM recruitment and reprogramming of these cells^[37,38]^, particularly when combined with other immunotherapies^[39]^. In thyroid cancer, the effects of CSF1R inhibition by PLX3397 and inhibition of Spp1 have been noted^[40]^; however, the specific signaling pathways remain to be elucidated. In general, mechanistic studies are warranted to further elucidate how R848 and PLX3397 contribute to Spp1 down-regulation in TAM.

To enhance the targeted delivery of small molecules to TAM after systemic injection, we used a cyclodextrin-adjuvant nanoparticle-drug delivery system^[28,32,41,42]^. Earlier iterations of this platform were engineered to induce IL-12^[28,42]^ or CXCL9 in TAM^[32]^. Since prior scRNAseq data had shown mutually exclusive CXCL9 and SPP1 expression in TAM (**Fig. S1** ^[1]^), we initially tested whether the CXCL9-modulating CANDI preparation (CANDI400) could affect Spp1 levels. However, we observed only minor effects, prompting us to design the screening strategy featured here.

Limitations and opportunities for future research

While this study successfully designed an efficient small molecule-based approach for silencing Spp1 in macrophages, several limitations warrant consideration. First, the use of bone marrow-derived macrophages from donor mice restricted the scope of drugs, doses, and combinations we could realistically test. Additional formulations and combinations may have similar or even stronger effects, and therapeutics beyond small molecules may also prove effective in silencing Spp1 phenotypes. Second, we started with a relatively small library of compounds, each purported to inhibit SPP1 through different pathways. There is potential to perform unbiased screens of larger compound libraries, which could reveal new compounds with similar or new mechanisms of action. Third, our screening focused on Spp1 inhibition. For enhanced in vivo efficacy, it may be advantageous to combine these compounds with others that robustly induce immunostimulatory pathways, such as CXCL9 or IL-12. The modular nature of the CANDI platform supports this possibility though it will require further refinement to optimise drug loading and combinations. Despite some of these limitations, our initial results are highly encouraging, suggesting the potential for developing a new class of therapeutics targeting myeloid cells. These therapeutics aim to polarize TAM towards an Spp1^Low^ phenotype, thereby enhancing anti-tumor immunity. Further research and fine-tuning will be crucial in optimizing these strategies and exploring their full therapeutic potential.

## Materials and Methods

**Materials**

All reagents and solvents were obtained from Thermo Fisher or Sigma-Aldrich and used without additional purification. The small molecules (**Fig. S3**), including Lificiguat, IDF1174, CAY10585, Chrysin, OPN expression inhibitor 1, Andrographolide, Mesalazine, Entrectinib, CPSI-1306, Iguratimod, Losartan, Parecoxib, Troglitazone, Indomethacin, Resiquimod, PLX5622, PLX3397, Shikonin, RN-1734, Trypthantrin, GSK-3484862, Procainamide, Tofacitinib, Baricitinib, and Curcumin, were purchased from MedChemExpress. The compounds were dissolved in dimethyl sulfoxide (DMSO) as needed and were used without further treatment. MilliQ water was sourced from the Waters filtration system.

**Particle synthesis**

We synthesized sbCD from scratch with a defined degree of substitution of 2.5^[28,43]^, to address the variability and high cost associated with commercially available reagents. This sbCD was used to create small nanoparticles with an average diameter of 16 nm (polydispersity index, PDI=0.198). To obtain crosslinked bsCD nanoparticles (CANDI), L-lysine was added dropwise to sbCD, and the reaction mixture was stirred for 18 hours. The resulting particles were precipitated using ice-cold ethanol, then purified and characterized using DLS and Zeta potential analysis before being stored at -20 °C^[28]^.

Fluorescent CANDI Analogs. Lyophilized CANDIE was dissolved in a 0.1 M carbonate buffer at pH 8.5, and AF647 succinimidyl ester (ThermoFisher, 2 mg/mL in DMSO) was added to reach a concentration of 50 μM. The mixture was stirred at 37°C for 45 minutes in a thermocycler at 600 rpm. The labeled nanoparticles were then purified using buffer exchange with water, passing through 10 kDa MWCO centrifugal filters (Amicon; 10,000 rpm for 5 minutes; 300 μL water per wash, repeated 4−5 times). The final products were diluted with water or PBS to a concentration of 50 mg/mL and filtered through a 0.22 μm sterile filter (VWR) before use.

**Characterization**

The particle size and surface charge of all nanoparticle formulations was measured using dynamic light scattering (DLS) and zeta potential analysis on a Malvern Zetasizer APS. Measurements were conducted at a concentration of 2 mg/mL in PBS using DTS1170 cuvettes (Malvern) at 25 °C.

Small-molecule loading of nanoparticles (**Table S1**).

For payload loading, a solution of empty CANDI (5 mg; **Table S3**) in 90 μL of PBS (1x) was prepared in 10% DMSO. The following nanoparticle formulation was developed: CANDI460 containing R848 (0.25 mg) and PLX3397 (0.142 mg). 10 μL of 2M NaOH and 20 μL of 1M HCl were added, and the mixtures were rapidly vortexed until the drugs were fully dissolved. The solution was then filtered through a 0.22 μm sterile filter (VWR) and either used immediately for characterization and in vitro assays or stored at -20 ºC for later use.

Turbidity assay.

This assay was used to determine drug loading. CANDIE solutions (0.39-100 mg/mL in 1x PBS) were prepared at pH 7.4, and small molecules were dissolved in DMSO (0.0638 mg R848 and 0.0362 mg PLX3397 per well) to create payload stocks. The payloads were then added to buffered CANDI. Loading efficiency was assessed by measuring absorbance at 550 nm (λabs) after thorough mixing. Complete absorbance loss indicated full payload loading. Data were normalized against a payload-free control, with all experiments conducted in triplicate (N = 3).

Drug Release Kinetics. The kinetics of drug release were evaluated using a closed dialysis setup with a 3 kDa molecular weight cutoff membrane (Pur-A-Lyzer Midi Dialysis Kit). Solutions of CANDI400 (50 mg) were prepared with PLX3397 (0.56 mM) and R848 (1.3 mM) in 1 mL of phosphate-buffered saline (PBS, 1×) containing 10% dimethyl sulfoxide (DMSO). These solutions were dialyzed against 5 mL of DMSO containing 20% PBS (1×) at 37°C, under constant stirring at 600 rpm. At predetermined time points, 100 μL aliquots were withdrawn from the dialysis setup and analyzed using liquid chromatography-mass spectrometry (LC-MS). Each aliquot was replaced with an equal volume of fresh DMSO (20% PBS) to maintain constant volume. The identification of PLX3397 and R848 was based on their characteristic retention times (0.92 min and 0.78 min, respectively) and their mass-to-charge ratios (PLX3397: 421, R848: 315) in positive electrospray ionization mode (ES+). The percentage of eluted molecules was quantified by integrating the area under the curve (AUC) for each chromatographic peak, and the cumulative drug release was calculated as the ratio of the AUC of each eluted peak to the total AUC of chromatographs obtained from non-dialyzed solutions. All experiments were conducted in triplicates (N = 3)

Transmission Electron Microscopy. CANDI460 particles were prepared (50 mg mL^-1^, PBS 1x) and diluted with water to a final concentration of 0.1 mg mL^-1^. The particle solution was charged on a TEM grid for 1 min and treated with a 2% aqueous uranyl acetate solution for 15 min, followed by three washing steps with ultra-pure water (x3). Imaging was performed in a transmission electron microscope (JEOL 2100).

**In vitro experiments**

Immortalized cell lines. The immortalized murine bone marrow-derived macrophages (iMACs) used to evaluate toxicity were obtained from Charles L. Evavold at the Ragon Institute, Harvard University, as described by Evavold et al. (2021, Cell, 184, 4495). The iMAC and MC38 cells were cultured in Dulbecco’s Modified Eagle Medium (DMEM, Corning) with 10% Fetal Bovine Serum (Corning) and 1% Penicillin Streptomycin (Corning) at 37°C with 5% CO2. When the cells reached confluency, they were split using 0.05% Trypsin / 0.53 mM EDTA (Corning), and all in vitro assays were conducted when cells were 90% confluent. Before being used in cell culture, all CANDI preparations were filtered through a 0.22 μm sterile filter (VWR).

Bone marrow-derived cells. Murine bone marrow-derived cells (BMDCs) were isolated from Spp1-tdTomato reporter mice and wild-type C57BL/6J mice (see below). BMDCs from the reporter mice were used for flow cytometry and live-cell microscopy, while those from wild-type mice were used for cytokine induction evaluation. To collect bone marrow, femurs were flushed with sterile PBS using syringes and a 27-gauge needle. Red blood cells were lysed using RBC Lysis Buffer (BioLegend) as per the manufacturer’s guidelines. The remaining cells were counted with a Neubauer chamber and plated into 96-well plates—transparent plates (NEST) for flow cytometry or black plates (ibidi, glass bottom) for imaging—at a density of 1.1 x 10^5 cells per well. Bone marrow-derived macrophages (BMDMs) were differentiated by adding 50 ng/mL recombinant murine M-CSF (BioLegend) to the culture media over 5 days. The treatment was applied on day 1 for 24 hours, with fresh media added on day 4. For RNA sequencing, cells were plated into transparent 6-well plates (Corning) at a density of 1 x 10^6 cells per well. Cells were stimulated with 50 ng/mL recombinant murine M-CSF (BioLegend) for 7 days before treatment. Fresh media was added on day 4. RNA was purified with the RNeasy Mini Kit (Qiagen) according to the manual.

Live-Cell Microscopy. Cells were exposed to a range of small molecules (0−1 μM, DMSO < 0.5%) for 24 hours by adding pre-prepared stock solutions to the culture media. Prior to imaging, cells were stained with Hoechst 33342 (15 μg/mL, Thermo Fisher) following the manufacturer’s instructions. Imaging was conducted in a 96-well plate using an IX81 inverted fluorescence microscope (Olympus, Tokyo, Japan) equipped with a motorized stage (Renishaw, Wotton-under-Edge, England, UK) and an ORCA-Fusion Digital CMOS camera (Hamamatsu Photonics, Hamamatsu, Japan). Multiple fields of view were captured for each sample with either a UPlanSApo ×10 objective (NA 0.75, Olympus) or a UPlanSApo 40× air objective (NA 0.95, Olympus), using CellSens Dimension 3.1.1 software (Olympus). Along with bright-field images, two fluorescent channels were recorded: DAPI (345/455) and TdTomato (550/585), using the corresponding optical filters for excitation. Images were analyzed with the Cell Profiler (<https://cellprofiler.org>), and fluorescence data was expressed on a scale from 0 to 1.

Flow Cytometry. Bone-marrow-derived cells from Spp1-TdTomato reporter mice were stimulated with specific drug combinations, then trypsinized and washed with PBS. The cells were first stained with AquaAmine LiveDead Fixable viability stain (Thermo Fisher) diluted in PBS, followed by treatment with Fc block (BioLegend) and fluorochrome-conjugated antibodies (Table S3) prepared in FACS buffer (1× PBS, 2 mM EDTA, 2% FBS). For flow cytometry analysis, the cells were resuspended in FACS buffer. All samples were measured using an Attune NxT flow cytometer (Thermo Fisher), and the data was analyzed with FlowJo 10 software (TreeStar).

Toxicity. iMACs were seeded in 96-well plates at a density of 15 x 10^3 cells per well and incubated for 24 hours at 37 °C with 5% CO2 before use. A stock solution of CANDI460 was prepared and diluted in cell culture medium to the desired concentrations (0.153 μg/mL to 10 mg/mL, DMSO 0.5%). Cells were incubated with nanoparticles for 24 hours, after which the medium was replaced with FluoroBrite DMEM containing AlamarBlue (Invitrogen; 10% final). Cells were then incubated at 37 °C and 5% CO2 for an additional hour. The fluorescence of each well was measured (λex = 550 nm, λem = 590 nm). Triplicates were performed for each concentration tested, and IC50 values were calculated from the means.

Dose response. To assess the dose-response of the dual-labeled nanoparticle, a stock solution of CANDI460 nanoparticles (Table S2) was prepared and then diluted in cell culture medium to achieve the desired concentrations (6.1 ng/mL to 1.6 mg/mL, DMSO 0.5%). Spp1-tdTomato BMDM reporter cells were incubated with the nanoparticle-spiked media on day 1 for 24 hours, with fresh media added on day 4. The cells were then prepared for fluorescence microscopy to determine Spp1-dtTomato expression.

**In vivo experiments**

Mouse models. All animals were bred and housed under specific pathogen-free conditions at the Massachusetts General Hospital. Experiments were approved by the MGH Institutional Animal Care and Use Committee (IACUC) and were performed in accordance with MGH IACUC regulations. Table S1 summarizes mouse models and numbers. The main model was the Spp1-IRES-tdTomato mouse, a CRISPR/Cas9 generated mutant carrying a tdTomato reporter inserted after the stop codon in exon 7 of the Spp1 gene. The Spp1-IRES-TdTomato model is available as B6J.Spp1^tm1(tdTomato)Msasn/J^ (stock 33731) from the Jackson Laboratory. We also crossed Spp1-IRES-TdTomato mice with Mert-TK-GFP mice^[44]^ to define the intratumoral SPP1 cell population.

Tumor Cell Implantation. MC38 and B16−F10 cells were injected into the flanks of C57BL/6J mice at concentrations of 2.5 × 10^6^ cells and 0.5 × 10^6^ cells, respectively. The tumors were given a minimum of one week to develop before treatment began and reached a size of at least 50 mm³ before therapy was initiated.

Intravital microscopy. Imaging of mice bearing dorsal window chambers with MC38-mTAG-BFP or MC38-H2B-GFP was performed to determine the kinetics of Spp1 modulation in the tumor microenvironment (**Fig 5**). Dorsal window chambers were implanted into reporter mice using well-established techniques^[45]^. Fluorescent tumor cells (MC38-H2B-GFP or MC38-mTAG-BFP) were implanted in the window chambers as previously described^[46,47]^ and allowed to grow for 7-10 days before imaging experiments, with tumor growth monitored regularly. In additional experiments, we determined the vascular half-live of CANDI460 by serial imaging of the microvascular in the mouse ear.

All confocal images were collected using a customized Olympus FV1000 confocal microscope (Olympus America). A 2x (XLFluor, NA 0.14), a 4x (UPlanSApo, NA 0.16), and an XLUMPlanFL N 20x (NA 1.0) water immersion objective were used for imaging (Olympus America). Fusion-protein MC38-Tag2-BFP, macrophage host cells (MerTK-GFP), fusion-protein Spp1-tomato and CANDI^AF647^ were excited sequentially using a 405 nm, a 473 nm, a 559 nm, and a 633 nm diode laser in combination with a DM-405/488/559/635 nm dichroic beam splitter. Emitted light was further separated by beam splitters (SDM-473, SDM-560, and SDM-640) and emission filters BA430-455, BA490-540, BA575-620, and BA655-755 (Olympus America). Confocal laser power settings were carefully optimized to avoid photobleaching, phototoxicity, or tissue damage. Fiji (ImageJ, 2.9.0/1.53t) was used for image analysis.

Flow cytometry. Mice with MC38 tumors were treated with one dose of CANDI460, and tumors were harvested after 4 days. Subsequently, the tumors were processed in RPMI medium with 0.2 mg/ml and then passed through 40µm filters. Next, the cells were stained with AquaAmine LiveDead Fixable viability stain (Thermo Fisher), which was diluted in PBS. Following this, they were treated with an Fc block (BioLegend) and then stained with fluorochrome-conjugated antibodies (**Tables S3**), which were diluted in FACS buffer (1x PBS, 2 mM EDTA, 2% FBS). For flow cytometry analysis, the cells were resuspended in FACS buffer. Each condition was assessed in triplicate or quadruplicate using an Attune NxT flow cytometer (Thermo Fisher), and the resulting data were analyzed with FlowJo 10 software (TreeStar).

Drug treatment. CANDI460 was administered via tail-vein injection using 100 µL of normal saline containing 5 mg of nanoparticles per mouse, loaded with 0.25 mg of R848 and 0.142 mg of PLX3397. Before injection, the solution was sterilized using a 0.22 μm sterile centrifugal filter (VWR), vortexed, and used immediately. Drug treatments generally involved 3 separate injections as detailed in **Fig 4** and **Fig S12.**

Histology

MC38-WT tumors were harvested and fixed in 10% formalin solution. The tumors were paraffin-embedded and sectioned at 5 µm. The sections were then deparaffinized and rehydrated before immunofluorescence staining. Heat-induced antigen retrieval was performed using Retrievagen A pH6.0 (550524, BD Bioscience), and the sections were permeabilized with 0.3% Triton X-100 in PBS for 10 minutes at room temperature. After the sections were blocked with 4% normal goat serum in PBS, Spp1 antibody (EPR21138; ab218237, Abcam 1:100) was incubated at 4°C overnight. The sections were incubated with a biotinylated goat anti-rabbit IgG antibody followed by streptavidin DyLight 594 (BA-1000 and SA-5594, Vector Laboratories, 1:100 and 1:600 respectively), and the nuclei were stained with DAPI (D21490, Thermo Fisher Scientific 1:3000). After Spp1 staining, the coverslips were removed and H&E staining was performed according to manufacturer’s protocol (ab245880, Abcam). All the slides were scanned using the NanoZoomer 2.0RS scanner (Hamamatsu) for analysis.

**Statistical analysis**

All statistical data analyses were performed using GraphPad Prism 10 software, and results are expressed as mean ± standard deviation. We used a 2-tailed Student’s t-test and one-way ANOVA followed by Bonferroni’s multiple comparison tests for normally distributed datasets. We performed non-parametric Mann-Whitney or Kuskal-Wallis tests when variables were not normally distributed. For survival analysis, p values were computed using the Log Rank test. p values > 0.05 were considered insignificant (n.s.), and p values < 0.05 were considered significant. ∗ p value < 0.05, ∗∗ p value < 0.01, ∗∗∗ p value < 0.001, ∗∗∗∗ p value < 0.0001.

**Acknowledgments**

We thank members of CSB for many helpful discussions, Drs. Charles Evavold for the kind gift of the IMAC line. This work was partly supported by R01CA281735, R01CA257623, R33CA277820 and the CSB Development Fund. BK was supported by the Studienstiftung (Germany). EAH was previously supported by the Swiss National Science Foundation (P2EZP2_191831).

**Contributions**

Conceptualization: MP, RW

Data curation: BK, HSK, AGG, RK

Formal analysis: all authors

RNAseq analysis: YX, PW, CSG

Methodology: CSG, MP, RW,

Modeling: JC

Validation: CSG, HSK, EAH, RW

Supervision: CSG, MP, RW

Visualization: RW

Writing - original draft: RW

Writing - review and editing: RW and all coauthors

Funding acquisition: RW

Project administration: RW

Resources: RW

**ORCID**

Benan Kartal: 0009-0001-3565-2311

Christopher S. Garris: 0000-0002-5764-6203

Hyung Shikh Kim: 0000-0002-4209-2471

Anne-Gaëlle Goubet: 0000-0003-3431-3067

Halabi Elias A: 0000-0003-1910-8196

Yoshiko Iwamoto: 0000-0002-3723-1043

Yuxuan Xie: 0009-0008-0640-2269

Pratyaksha Wirapati: 0000-0002-0327-4686

Rainer H. Kohler: 0000-0001-6269-6590

Mikaël J. Pittet: 0000-0002-2060-4691

Ralph Weissleder: 0000-0003-0828-4143

**Conflict of interest**

RW is a consultant to Boston Scientific, Earli, and Accure Health, none of whom contributed to this research. MJP has consulted for AstraZeneca, Elstar Therapeutics, ImmuneOncia, KSQ Therapeutics, Merck, Siamab Therapeutics, Third Rock Ventures, and Tidal. The other authors report no affiliations.

## References

[1] R. Bill, P. Wirapati, M. Messemaker, W. Roh, B. Zitti, F. Duval, M. Kiss, J. C. Park, T. M. Saal, J. Hoelzl, D. Tarussio, F. Benedetti, S. Tissot, L. Kandalaft, M. Varrone, G. Ciriello, T. A. McKee, Y. Monnier, M. Mermod, E. M. Blaum, I. Gushterova, A. L. K. Gonye, N. Hacohen, G. Getz, T. R. Mempel, A. M. Klein, R. Weissleder, W. C. Faquin, P. M. Sadow, D. Lin, S. I. Pai, M. Sade-Feldman,M. J. Pittet. *Science* **2023**, *381*, 515.

[2] M. J. Pittet, O. Michielin,D. Migliorini. *Nat. Rev. Clin. Oncol.* **2022**, *19*, 402.

[3] Y. Du, Y. Lin, L. Gan, S. Wang, S. Chen, C. Li, S. Hou, B. Hu, B. Wang, Y. Ye,Z. Shen. *J Transl Med* **2024**, *22*, 158.

[4] S. M. Hong, A. Y. Lee, B. J. Kim, J. E. Lee, S. Y. Seon, Y. J. Ha, J. T. Ng, G. Yoon, S. B. Lim, M. J. Morgan, J. H. Cha, D. Lee,Y. S. Kim. *Adv Sci (Weinh)* **2024**, e2303177.

[5] N. Li, Q. Zhu, Y. Tian, K. J. Ahn, X. Wang, Z. Cramer, J. Jou, I. W. Folkert, P. Yu, S. Adams-Tzivelekidis, P. Sehgal, N. N. Mahmoud, C. B. Aarons, R. E. Roses, A. Thomas-Tikhonenko, E. E. Furth, B. Z. Stanger, A. Rustgi, M. Haldar, B. W. Katona, K. Tan,C. J. Lengner. *Nat Commun* **2023**, *14*, 7915.

[6] D. Lin, T. Zheng, S. Huang, R. Liu, S. Guan,Z. Zhang. *Sci Rep* **2024**, *14*, 2767.

[7] H. Luo, X. Xia, L. B. Huang, H. An, M. Cao, G. D. Kim, H. N. Chen, W. H. Zhang, Y. Shu, X. Kong, Z. Ren, P. H. Li, Y. Liu, H. Tang, R. Sun, C. Li, B. Bai, W. Jia, Y. Liu, W. Zhang, L. Yang, Y. Peng, L. Dai, H. Hu, Y. Jiang, Y. Hu, J. Zhu, H. Jiang, Z. Li, C. Caulin, J. Park,H. Xu. *Nat Commun* **2022**, *13*, 6619.

[8] J. Qi, H. Sun, Y. Zhang, Z. Wang, Z. Xun, Z. Li, X. Ding, R. Bao, L. Hong, W. Jia, F. Fang, H. Liu, L. Chen, J. Zhong, D. Zou, L. Liu, L. Han, F. Ginhoux, Y. Liu, Y. Ye,B. Su. *Nat Commun* **2022**, *13*, 1742.

[9] E. P. Storrs, P. Chati, A. Usmani, I. Sloan, B. A. Krasnick, R. Babbra, P. K. Harris, C. M. Sachs, F. Qaium, D. Chatterjee, C. Wetzel, S. P. Goedegebuure, T. Hollander, H. Anthony, J. Ponce, A. M. Khaliq, S. Badiyan, H. Kim, D. G. Denardo, G. D. Lang, N. D. Cosgrove, V. M. Kushnir, D. S. Early, A. Masood, K. H. Lim, W. G. Hawkins, L. Ding, R. C. Fields, K. K. Das,A. A. Chaudhuri. *NPJ Precis Oncol* **2023**, *7*, 105.

[10] E. Matsubara, H. Yano, C. Pan, Y. Komohara, Y. Fujiwara, S. Zhao, Y. Shinchi, D. Kurotaki,M. Suzuki. *Cancers (Basel)* **2023**, *15*, 2250.

[11] M. Hulsmans, M. J. Schloss, I. H. Lee, A. Bapat, Y. Iwamoto, C. Vinegoni, A. Paccalet, M. Yamazoe, J. Grune, S. Pabel, N. Momin, H. Seung, N. Kumowski, F. E. Pulous, D. Keller, C. Bening, U. Green, J. K. Lennerz, R. N. Mitchell, A. Lewis, B. Casadei, O. Iborra-Egea, A. Bayes-Genis, S. Sossalla, C. S. Ong, R. N. Pierson, J. C. Aster, D. Rohde, G. R. Wojtkiewicz, R. Weissleder, F. K. Swirski, G. Tellides, G. Tolis, S. Melnitchouk, D. J. Milan, P. T. Ellinor, K. Naxerova,M. Nahrendorf. *Science* **2023**, *381*, 231.

[12] D. V. F. Tauriello, E. Sancho,E. Batlle. *Nat Rev Cancer* **2022**, *22*, 25.

[13] M. Bandopadhyay, A. Bulbule, R. Butti, G. Chakraborty, P. Ghorpade, P. Ghosh, M. Gorain, S. Kale, D. Kumar, S. Kumar, K. V. Totakura, G. Roy, P. Sharma, D. Shetti, G. Soundararajan, D. Thorat, D. Tomar, R. Nalukurthi, R. Raja, R. Mishra, A. S. Yadav,G. C. Kundu. *Expert Opin Ther Targets* **2014**, *18*, 883.

[14] N. Momin, S. Pabel, A. Rudra, N. Kumowski, I.-H. Lee, K. Mentkowski, M. Yamazoe, L. Stengel, C. G. Muse, H. Seung, A. Paccalet, C. Gonzalez-Correa, E. B. Jacobs, J. Grune, M. J. Schloss, S. Sossalla, G. Wojtkiewicz, Y. Iwamoto, P. McMullen, R. N. Mitchell, P. T. Ellinor, D. G. Anderson, K. Naxerova, M. Nahrendorf,M. Hulsmans. *bioRxiv* **2024**, 2024.08.10.607461.

[15] S. De Schepper, J. Z. Ge, G. Crowley, L. S. S. Ferreira, D. Garceau, C. E. Toomey, D. Sokolova, J. Rueda-Carrasco, S. H. Shin, J. S. Kim, T. Childs, T. Lashley, J. J. Burden, M. Sasner, C. Sala Frigerio, S. Jung,S. Hong. *Nat Neurosci* **2023**, *26*, 406.

[16] C. Steidl, T. Lee, S. P. Shah, P. Farinha, G. Han, T. Nayar, A. Delaney, S. J. Jones, J. Iqbal, D. D. Weisenburger, M. A. Bast, A. Rosenwald, H. K. Muller-Hermelink, L. M. Rimsza, E. Campo, J. Delabie, R. M. Braziel, J. R. Cook, R. R. Tubbs, E. S. Jaffe, G. Lenz, J. M. Connors, L. M. Staudt, W. C. Chan,R. D. Gascoyne. *N Engl J Med* **2010**, *362*, 875.

[17] J. G. Quatromoni,E. Eruslanov. *Am J Transl Res* **2012**, *4*, 376.

[18] S. P. Arlauckas, C. S. Garris, R. H. Kohler, M. Kitaoka, M. F. Cuccarese, K. S. Yang, M. A. Miller, J. C. Carlson, G. J. Freeman, R. M. Anthony, R. Weissleder,M. J. Pittet. *Sci Transl Med* **2017**, *9*, eaal3604.

[19] M. De Palma,C. E. Lewis. *Cancer Cell* **2013**, *23*, 277.

[20] B. Z. Qian,J. W. Pollard. *Cell* **2010**, *141*, 39.

[21] X. Zhang, L. Peng, Y. Luo, S. Zhang, Y. Pu, Y. Chen, W. Guo, J. Yao, M. Shao, W. Fan, Q. Cui, Y. Xi, Y. Sun, X. Niu, X. Zhao, L. Chen, Y. Wang, Y. Liu, X. Yang, C. Wang, C. Zhong, W. Tan, J. Wang, C. Wu,D. Lin. *Nat Commun* **2021**, *12*, 5291.

[22] K. Pelka, M. Hofree, J. H. Chen, S. Sarkizova, J. D. Pirl, V. Jorgji, A. Bejnood, D. Dionne, W. H. Ge, K. H. Xu, S. X. Chao, D. R. Zollinger, D. J. Lieb, J. W. Reeves, C. A. Fuhrman, M. L. Hoang, T. Delorey, L. T. Nguyen, J. Waldman, M. Klapholz, I. Wakiro, O. Cohen, J. Albers, C. S. Smillie, M. S. Cuoco, J. Wu, M. J. Su, J. Yeung, B. Vijaykumar, A. M. Magnuson, N. Asinovski, T. Moll, M. N. Goder-Reiser, A. S. Applebaum, L. K. Brais, L. K. DelloStritto, S. L. Denning, S. T. Phillips, E. K. Hill, J. K. Meehan, D. T. Frederick, T. Sharova, A. Kanodia, E. Z. Todres, J. Jané-Valbuena, M. Biton, B. Izar, C. D. Lambden, T. E. Clancy, R. Bleday, N. Melnitchouk, J. Irani, H. Kunitake, D. L. Berger, A. Srivastava, J. L. Hornick, S. Ogino, A. Rotem, S. Vigneau, B. E. Johnson, R. B. Corcoran, A. H. Sharpe, V. K. Kuchroo, K. Ng, M. Giannakis, L. T. Nieman, G. M. Boland, A. J. Aguirre, A. C. Anderson, O. Rozenblatt-Rosen, A. Regev,N. Hacohen. *Cell* **2021**, *184*, 4734.

[23] S. R. Rittling. *Expert Rev Mol Med* **2011**, *13*, e15.

[24] Y. Liao, C. Wu, Y. Li, J. Wen,D. Zhao. *iScience* **2023**, *26*, 107273.

[25] J. Overgaard, J. G. Eriksen, M. Nordsmark, J. Alsner, M. R. Horsman,H. A. N. C. S. G. Danish. *Lancet Oncol* **2005**, *6*, 757.

[26] J. D. Klement, A. V. Paschall, P. S. Redd, M. L. Ibrahim, C. Lu, D. Yang, E. Celis, S. I. Abrams, K. Ozato,K. Liu. *J Clin Invest* **2018**, *128*, 5549.

[27] H. R. Moorman, Y. Reategui, D. B. Poschel,K. Liu. *Cells* **2022**, *11*, 2630.

[28] I. F. Fredrich, E. A. Halabi, R. H. Kohler, X. Ge, C. S. Garris,R. Weissleder. *ACS Nano* **2023**, *17*, 20666.

[29] J. Wei, Z. Chen, M. Hu, Z. He, D. Jiang, J. Long,H. Du. *Front Cell Dev Biol* **2021**, *9*, 749210.

[30] Y. Oyama, N. Akuzawa, R. Nagai,M. Kurabayashi. *Circ Res* **2002**, *90*, 348.

[31] C. S. Garris, S. P. Arlauckas, R. H. Kohler, M. P. Trefny, S. Garren, C. Piot, C. Engblom, C. Pfirschke, M. Siwicki, J. Gungabeesoon, G. J. Freeman, S. E. Warren, S. Ong, E. Browning, C. G. Twitty, R. H. Pierce, M. H. Le, A. P. Algazi, A. I. Daud, S. I. Pai, A. Zippelius, R. Weissleder,M. J. Pittet. *Immunity* **2018**, *49*, 1148.

[32] N. Enbergs, E. A. Halabi, A. G. Goubet, K. Schleyer, I. R. Fredrich, R. H. Kohler, C. S. Garris, M. J. Pittet,R. Weissleder. *Adv Sci (Weinh)* **2024**, e2309026.

[33] M. Ahmed, R. Behera, G. Chakraborty, S. Jain, V. Kumar, P. Sharma, A. Bulbule, S. Kale, S. Kumar, R. Mishra, R. Raja, S. Saraswati, R. Kaur, G. Soundararajan, D. Kumar, D. Thorat, M. Sanyal, A. Ramdasi, P. Ghosh,G. C. Kundu. *Expert Opin Ther Targets* **2011**, *15*, 1113.

[34] S. Kale, R. Raja, D. Thorat, G. Soundararajan, T. V. Patil,G. C. Kundu. *Oncogene* **2014**, *33*, 2295.

[35] J. Wei, A. Marisetty, B. Schrand, K. Gabrusiewicz, Y. Hashimoto, M. Ott, Z. Grami, L. Y. Kong, X. Ling, H. Caruso, S. Zhou, Y. A. Wang, G. N. Fuller, J. Huse, E. Gilboa, N. Kang, X. Huang, R. Verhaak, S. Li,A. B. Heimberger. *J Clin Invest* **2019**, *129*, 137.

[36] S. Kumar, H. S. Patil, P. Sharma, D. Kumar, S. Dasari, V. G. Puranik, H. V. Thulasiram,G. C. Kundu. *Curr Mol Med* **2012**, *12*, 952.

[37] S. M. Pyonteck, L. Akkari, A. J. Schuhmacher, R. L. Bowman, L. Sevenich, D. F. Quail, O. C. Olson, M. L. Quick, J. T. Huse, V. Teijeiro, M. Setty, C. S. Leslie, Y. Oei, A. Pedraza, J. Zhang, C. W. Brennan, J. C. Sutton, E. C. Holland, D. Daniel,J. A. Joyce. *Nat Med* **2013**, *19*, 1264.

[38] Y. Zhu, B. L. Knolhoff, M. A. Meyer, T. M. Nywening, B. L. West, J. Luo, A. Wang-Gillam, S. P. Goedegebuure, D. C. Linehan,D. G. DeNardo. *Cancer Res* **2014**, *74*, 5057.

[39] S. Hoves, C. H. Ooi, C. Wolter, H. Sade, S. Bissinger, M. Schmittnaegel, O. Ast, A. M. Giusti, K. Wartha, V. Runza, W. Xu, Y. Kienast, M. A. Cannarile, H. Levitsky, S. Romagnoli, M. De Palma, D. Rüttinger,C. H. Ries. *J Exp Med* **2018**, *215*, 859.

[40] S. Park, M. Kim, J. Zhu, W. K. Lee, G. Altan-Bonnet, P. Meltzer,S. Y. Cheng. *Am J Cancer Res* **2020**, *10*, 1857.

[41] C. B. Rodell, S. P. Arlauckas, M. F. Cuccarese, C. S. Garris, R. Li, M. S. Ahmed, R. H. Kohler, M. J. Pittet,R. Weissleder. *Nat. Biomed. Eng.* **2018**, *2*, 578.

[42] S. Lugani, E. A. Halabi, J. Oh, R. Kohler, H. Peterson, X. O. Breakefield, E. A. A. Chiocca, M. A. Miller, C. Garris,R. Weissleder. *Adv. Mater.* **2022**, *35*, e2208782.

[43] A. García, D. Leonardi,M. C. Lamas. *Bioorg. Med. Chem. Lett.* **2016**, *26*, 602.

[44] J. F. Mohan, R. H. Kohler, J. A. Hill, R. Weissleder, D. Mathis,C. Benoist. *Proc Natl Acad Sci U S A* **2017**, *114*, E7776.

[45] M. Alieva, L. Ritsma, R. J. Giedt, R. Weissleder,J. van Rheenen. *Intravital* **2014**, *3*, e29917.

[46] A. M. Laughney, E. Kim, M. M. Sprachman, M. A. Miller, R. H. Kohler, K. S. Yang, J. D. Orth, T. J. Mitchison,R. Weissleder. *Sci. Transl. Med.* **2014**, *6*, 261ra152.

[47] M. J. Pittet, C. S. Garris, S. P. Arlauckas,R. Weissleder. *Sci. Immunol.* **2018**, *3*, eaaq0491.

## Figures

**Fig. 1: SPP1 expression in tumor-associated macrophages (TAM). A.** TAM are often abundant and are recruited from the bone marrow via monocytes. SPP1 levels increase as monocytes differentiate into TAM. TAM can be targeted with systemically administered nanopreparations, a strategy that allows high local drug concentrations and multi-pharmacological pathway modulation. **B**. Note the negative correlation of TAM SPP1 with clinical outcomes in patients with HNSCC (correlation coefficient -0.85; p < 10^-5^)^[1]^. **C**. SPP1/Spp1 is induced by many different factors and conditions, usually pro-inflammatory conditions. The effects of Spp1 expression (osteopontin) and secretion are generally pro-tumorigenic and immunosuppressive. **D.** In vivo imaging of Spp1 in an MC38 mouse model. Note the abundance of Spp1-positive cells in the tumor microenvironment. **E.** Temporal Spp1 induction. Bone marrow cells were obtained from Spp1-TdTomato reporter mice and differentiated into macrophages using MCSF. The signal increases from 0.06 AU/cell to ~0.2 AU/cell and plateaus around 6-8 days. Representative Spp1-tdTomato images were obtained on day 1 (left), day 3 (middle), and day 5 (right). Note the increase in the Spp1 signal.


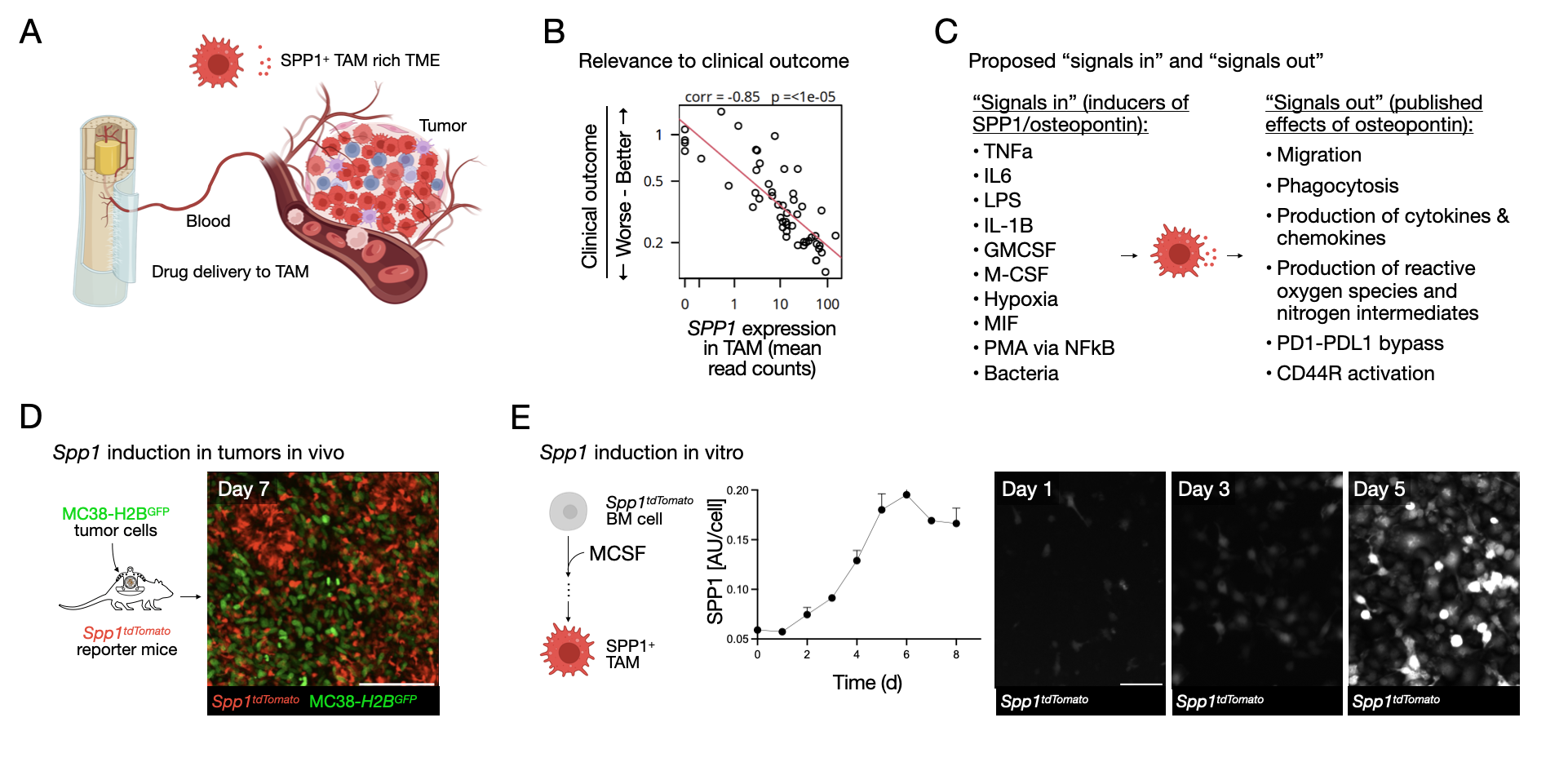


**Fig. 2: Small molecule screen to identify compounds capable of inhibiting Spp1 production. A.** Experimental outline. Bone marrow was harvested from Spp1-tdTomato reporter mice, and cells were differentiated into macrophages for 5 days. Cells were incubated with different concentrations (L: low, 0.1 µM; M: medium, 0.5 µM; H: high, 1 µM) of inhibitors on day 1, and Spp1 signal was determined on day 5. **B**. List of small molecule compounds tested. The list was chosen based on prior reports on pathway interactions of Spp1 (hypoxia^[29]^, MIF^[24]^, PPARG^[30]^ and putative direct inhibition. The color coding reflects normalized MFI as shown in A. Most putative Spp1 modulators did not affect the high Spp1 expression levels (red). Compounds that showed a dose-dependent effect (blue) included TLR agonists (R848), CSF1Ri (PLX3397 and PLX5622), and TNFa inhibitor (Shikonin). **C.** Dose-response of different small molecule modulators in decreasing SPP1. Bone marrow was harvested from Spp1-tdTomato reporter mice, and cells were differentiated into macrophages for 5 days. Cells were incubated with different concentrations of inhibitors on day 1, and SPP1 signal was determined on day 5. Note the Spp1 decrease as a function of drug dose. The green bar represents the approximate in vivo dose. Images on the right are representative examples of Spp1-tdTomato at baseline and at EC_50_. Scale bar: 100 µm. **D.** Drug effects (decrease in Spp1) for representative single, dually combined, or triple lead compounds. High Spp1 inhibition was observed with dual (85.8%) and triple( 91.4%) combinations. The graph on the right shows the cellular toxicity of the same small molecule combinations. Based on these data, we incorporated the top hits into CANDI460 for TAM delivery (**Fig. 4**).
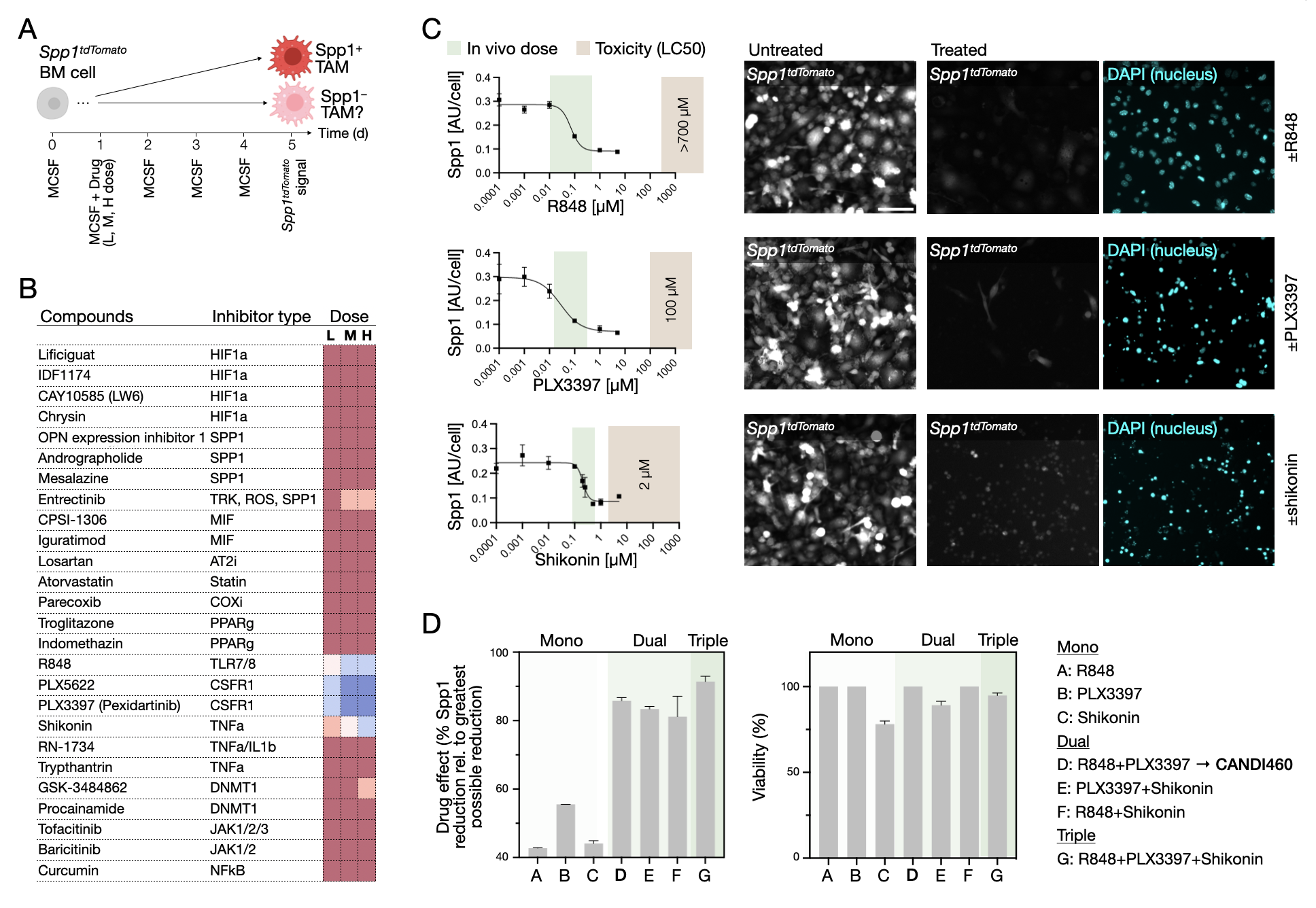


**Fig. 3: Characterization of triple-drug loaded CANDI460 nanoformulation.** **A**. CANDI was synthesized by cross-linking bis-succinyl cyclodextrin with lysine into ~16 nm nanoparticles. **B**. TEM of CANDi shows small uniform nanoparticles measuring ~18 nm in diameter. C. Payload of CANDI460. **D**. Biological characterization of CANDI460 in IMAC. Shown are dose-dependent cellular uptake and decrease of Spp1 as a function of dose (0.08 mg drug/mg CANDI). **E**. Cellular uptake of CANDI-460 (white) in Spp1-tdTomato expressing BMDM. Scale bar: 100µm. **F**. Cellular toxicity of CANDI460 in iMAC. The green shaded area shows the expected in vivo concentrations. Note the favorable therapeutic window. For high-resolution cellular uptake, see **Fig. S9**. **G**. Bulk RNAseq of BMDM incubated with CANDI460 vs empty CANDI. Note the down-regulation of Spp1 and up-regulation of CXCL9, and CCL5.

Fig 4


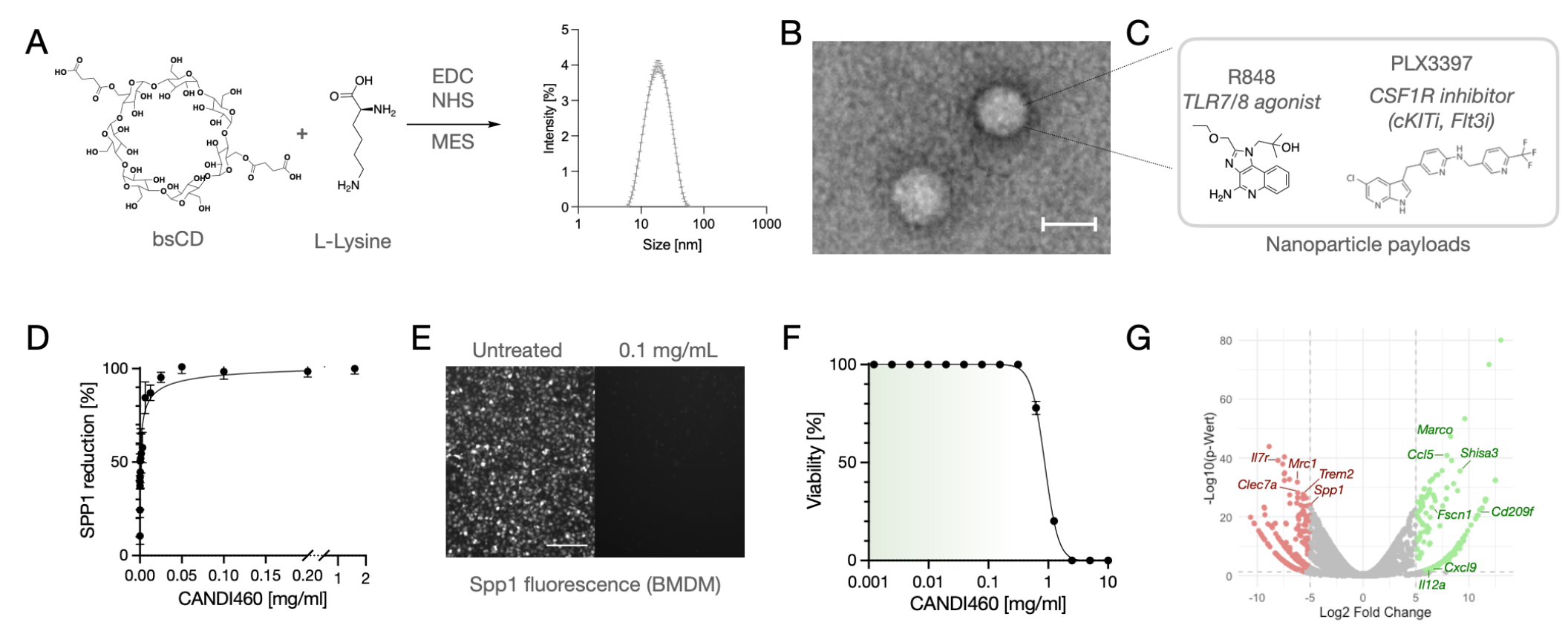


**Fig. 4: Efficacy in MC38 model. A.** MC38 tumors were implanted into the flank of mice on day 0. CANDI460 (5 mg/mouse) or control (CANDI empty) was administered IV on days 8, 10, 12 after tumor implantation (3 injections). Tumor sizes were monitored over time by caliper measurements. **B**. Tumor volume changes plotted as a function of time with three IV doses of CANDI460 or control. Note that of the 12 animals treated with CANDI460, 9 were tumor-free at 30 days. These tumor-free mice were resistant to MC38 re-challenge (**D**). Three of the 12 treated mice showed partial response to treatment. **C.** Survival curves. **D**. MC38 re-challenge experiment. Survivors (n=6) were reimplanted with MC38 after one month. **E**. Representative photographs of the tumor implantation site. H&E staining of MC38 tumors treated with CANDI460 or CANDI empty control. Note that no residual tumor exists in the representative example of this CANDI460-treated mouse. Scale bar: 100 µm.


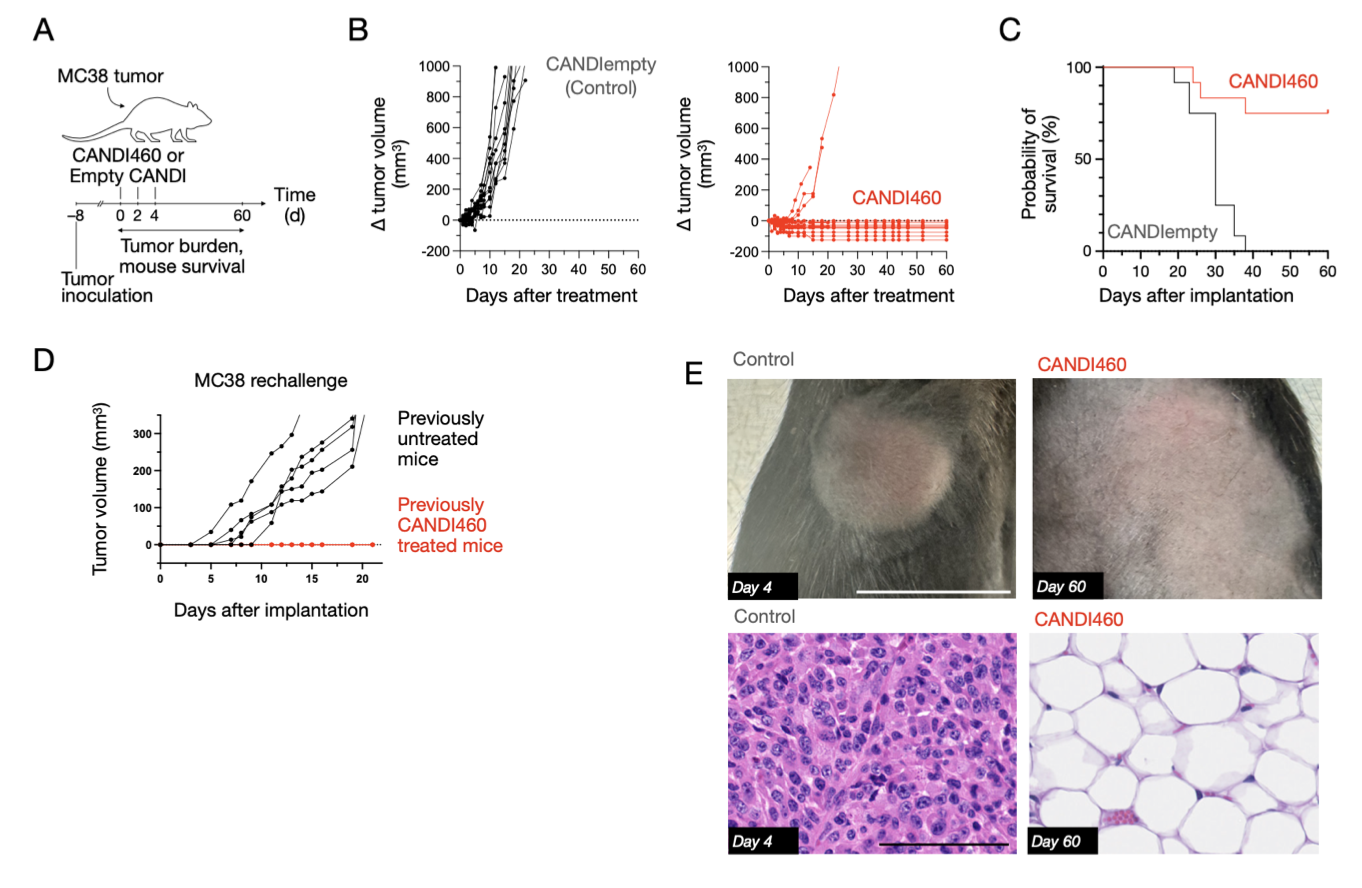


**Fig. 5: Serial In vivo microscopic imaging. A.** Dorsal window chamber model of MC38-H2BGFP tumors implanted in Spp1-tdTomato mice. Tumors were imaged 8 days after implantation when the MC38 tumors had grown into solid masses. At this time point (Day 0 pre), there was diffuse infiltration of the TME with highly Spp1-positive cells. **B-C**. Both the number (**B**)(p<0.0001) and the Spp1 mean fluorescence intensity (MFI, **C**) per cell (p<0.05) was higher than on subsequent days after treatment with an IV dose of CANDI460. Furthermore, tumors became smaller within 2 days of treatment (see also **Fig. S10** for temporal course). Scale bar: 100 µm.


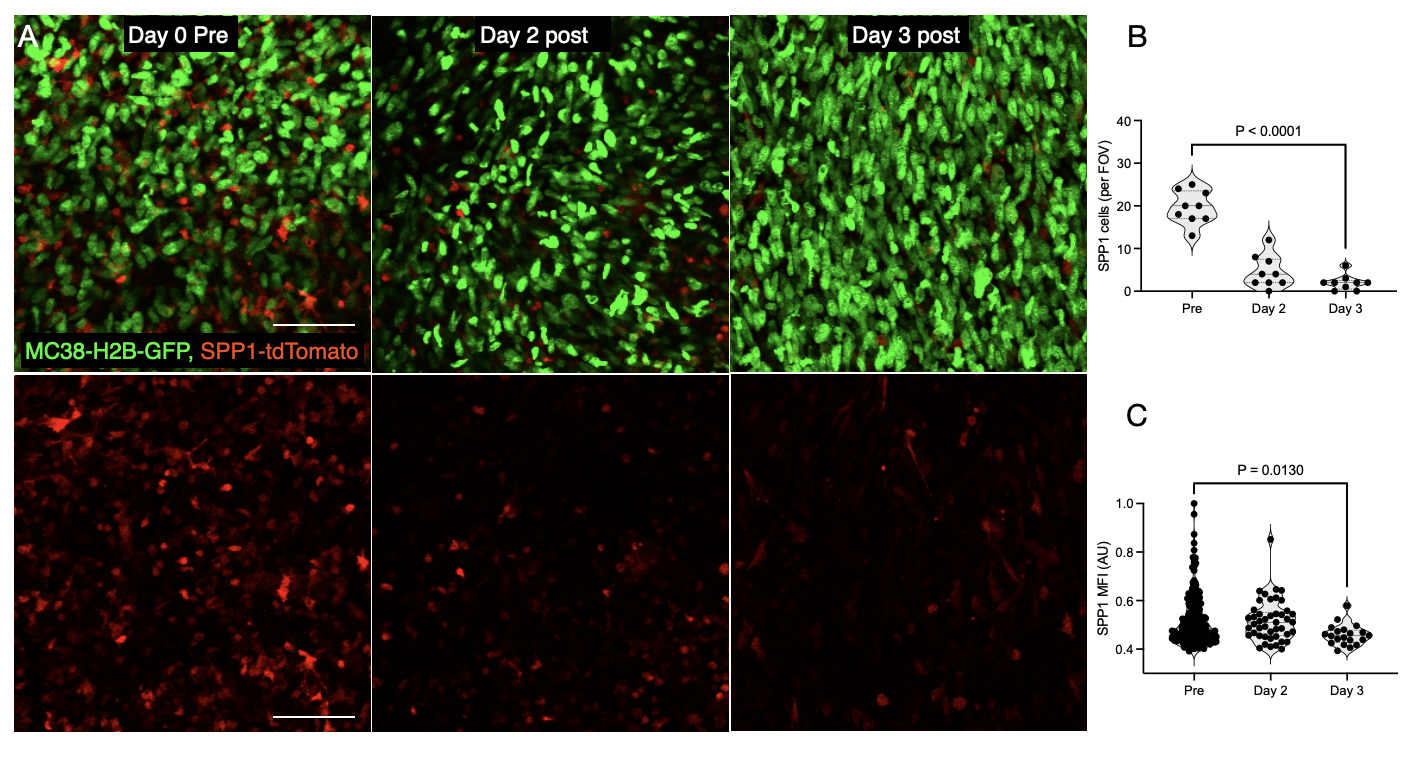


**Graphical abstract**

Spp1-producing tumor-associated macrophages (TAM) have been shown to herald an invariably negative outcome in many cancers due to their multipronged immunosuppressive role. We performed a screen to deliver small molecule SPP1-suppressing drugs via a CANDI nanoconstruct. With an IV injection, this counteracted SPP1-TAM and thus promoted cancer cures.


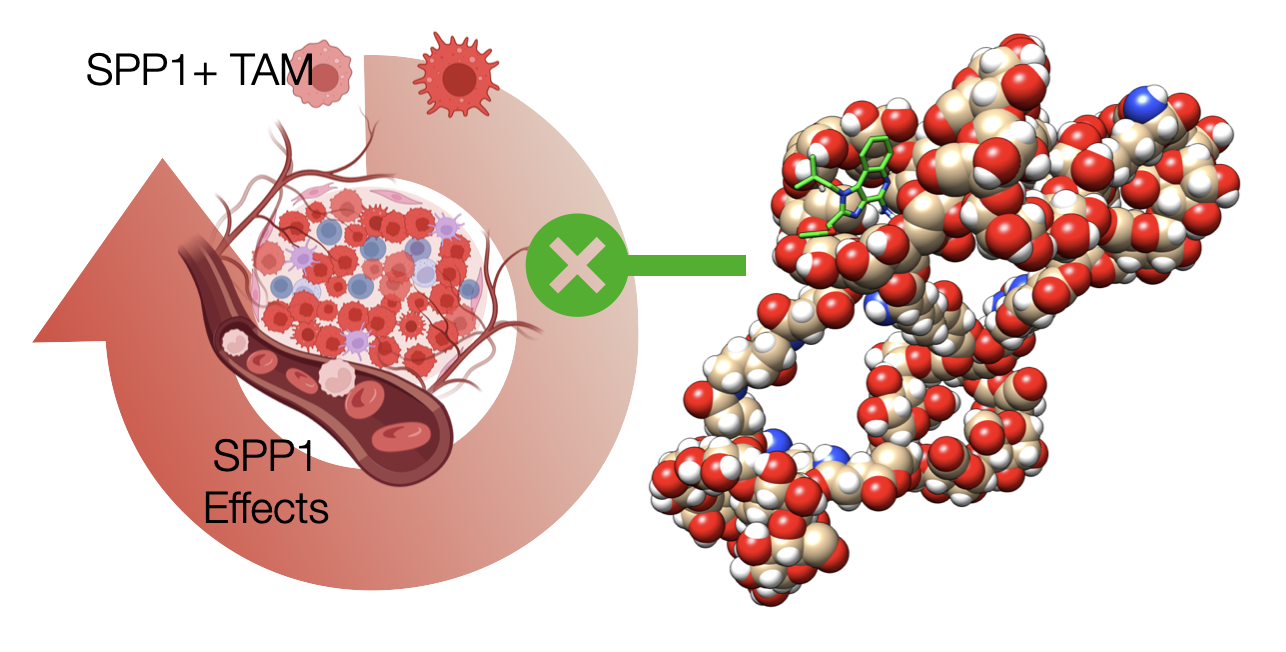


Supplemental information

**Targeted SPP1 inhibition of tumor-associated myeloid cells effectively decreases tumor sizes**

Benan Kartal^1^, Christopher S. Garris^1^, Hyung Shikh Kim^1^, Rainer Kohler^1^, Jasmine Carrothers^1^, Elias A. Halabi^1^, Yoshiko Iwamoto^1^, Anne-Gaëlle Goubet^2,3^, Yuxuan Xie, Pratyaksha Wirapati, Mikaël J. Pittet^2,3,4^, Ralph Weissleder^1,5,^*

^1^ Center for Systems Biology, Massachusetts General Hospital, 185 Cambridge St, CPZN 5206, Boston, MA 02114

^2^ Department of Pathology and Immunology, University of Geneva, Geneva, Switzerland

^3^ AGORA Cancer Research Center, Swiss Cancer Center Leman, Lausanne, Switzerland

^4^ Ludwig Institute for Cancer Research, Lausanne, Switzerland

^5^ Department of Systems Biology, Harvard Medical School, 200 Longwood Ave, Boston, MA 02115

*R. Weissleder, MD, PhD

Center for Systems Biology

Massachusetts General Hospital

185 Cambridge St, CPZN 5206

Boston, MA, 02114

617-726-8226

[rweissleder@mgh.harvard.edu](mailto:weissleder@helix.mgh.harvard.edu)

Keywords: nanoparticles, cancer, macrophage, SPP1

## Fig. S1: scRNAseq of SPP1 in human myeloid cells. Re-analysis of three previously published data sets. The data are for head and neck squamous cell cancer (from Bill et al, n = 52 patients^[1]^), colorectal cancer (from Pelka et al, n = 64 patients^[22]^) and esophageal squamous cell carcinoma (from Zhang et al, n = 58 patients^[21]^). Note the clear distinction between SPP1^Hi^ (red) and antitumorigenic CXCL9^Hi^ TAM (blue)


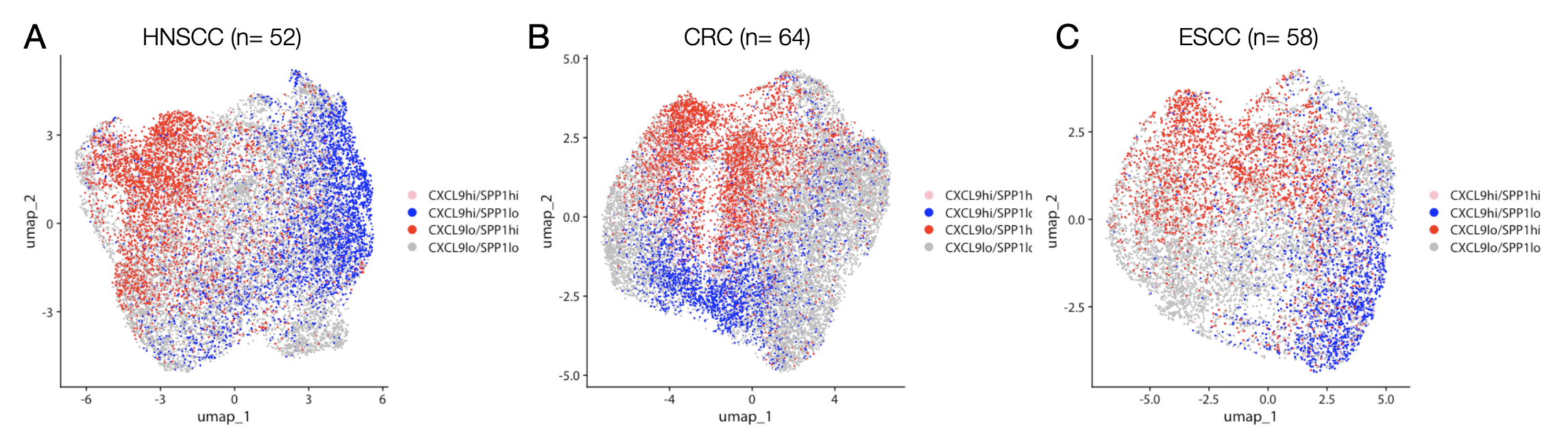


**Fig. S2: Therapeutic window of screen identified small molecule compounds.** Bone marrow was harvested from Spp1-tdTomato reporter mice, and cells were differentiated into macrophages for 4 days. Cells were then incubated with different concentrations of inhibitors, and Spp1 signal and cellular toxicity were determined 24 hours later.  **A**. TLR7/8 agonist R848 has the largest therapeutic window without apparent cellular toxicity at 100µM. The green bar represents the approximate in vivo dose, and the orange bar represents toxicity. **B**. Similar graph as in A but for PLX3397 (CSFR1 inhibitor). **C**. Data for Shikonin (indirect TNFa inhibitor) showing a narrow therapeutic window.


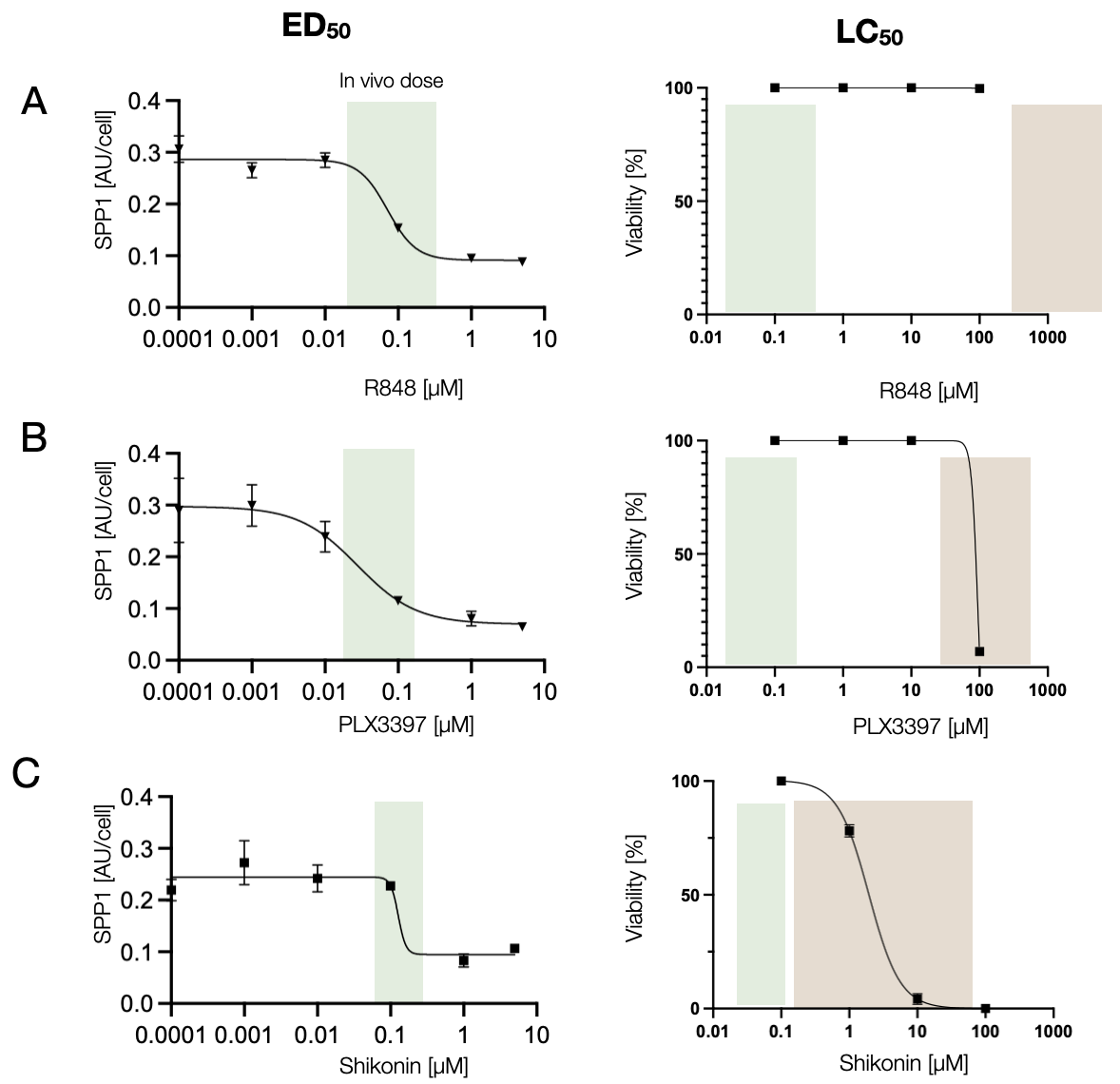


## Fig. S3: CANDI460 represents 18 nm nanoparticles crosslinked from bissuccinyl cyclodextrins which are loaded with R848 and PLX3397. B. Molecular model shows 5 units of bs-CD (out of ~1500 crosslinked units) and a single R848 C. Loading is possible in a dose-dependent fashion. On average, each mg of CANDI can hold 0.2 mg of small molecule drug load. D. Release of small molecule payloads plateaus after 7 hours

##
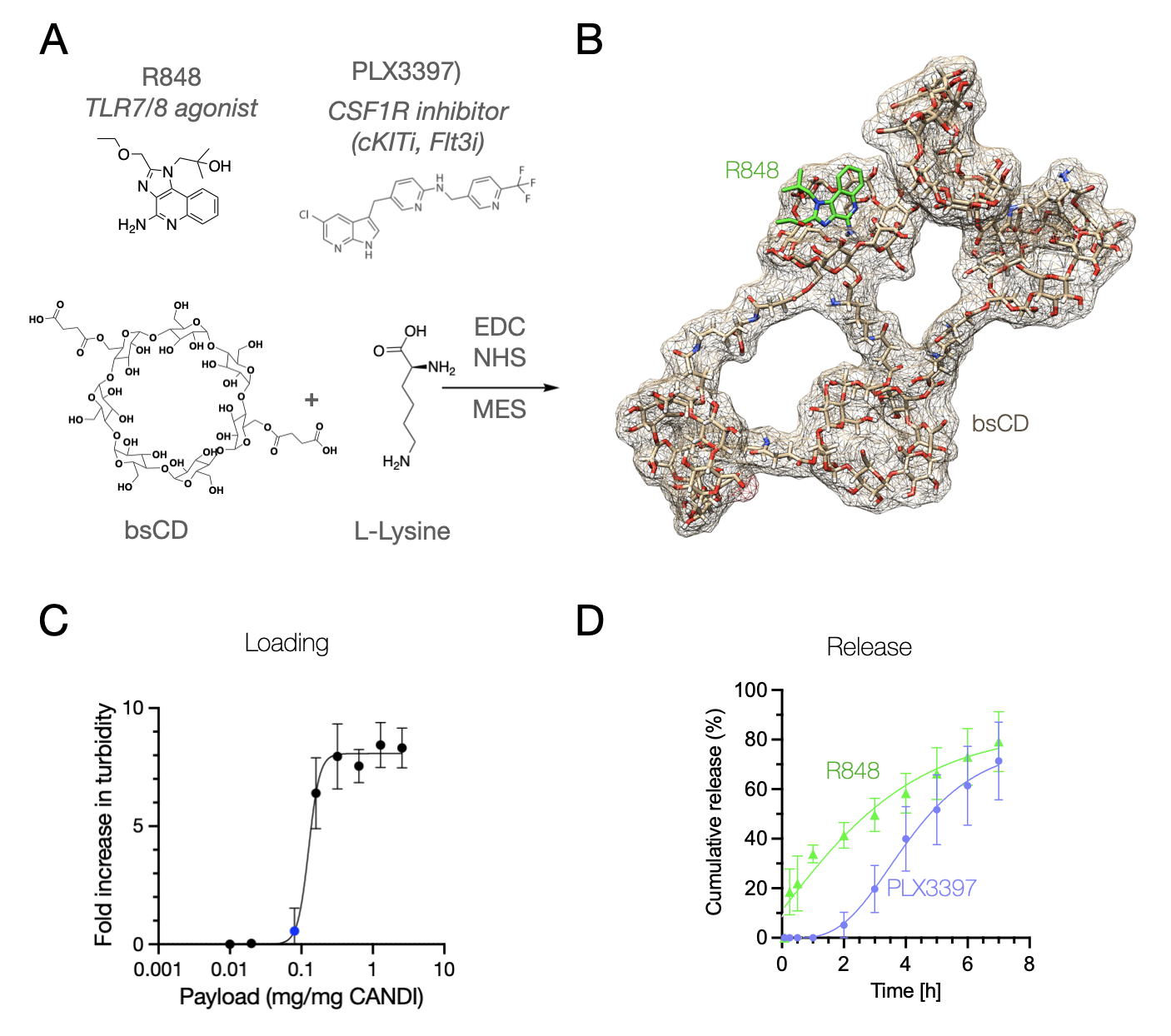


## Fig. S4: Modeling interaction of R848 with bs-CD. Wireframe (top) and ball and stick models (Bottom) of R848 (green) interacting with the bis-succinyl cyclodextrin ring

##
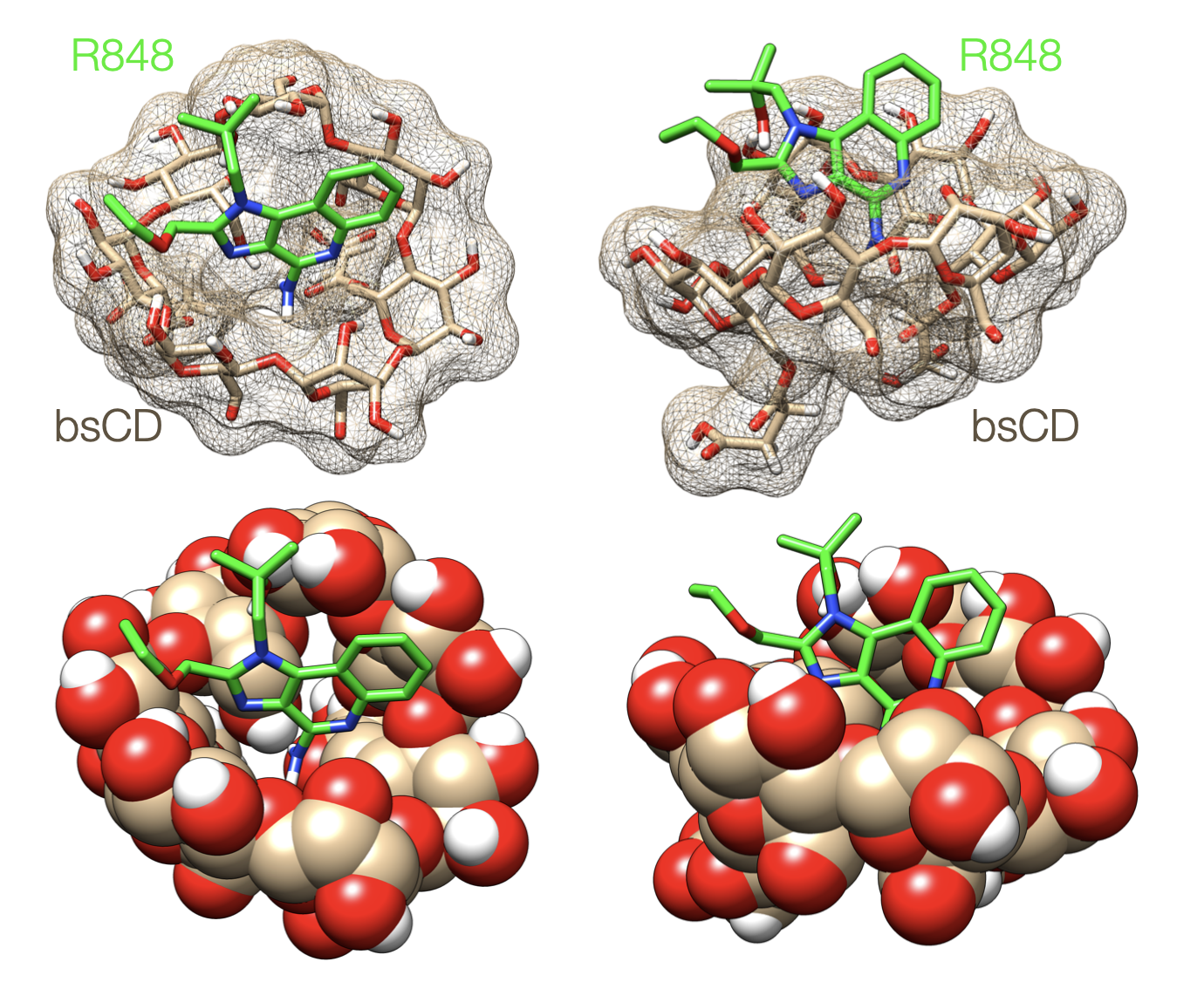


## Fig. S5: Modeling interaction of PLX3397 with bs-CD. Wireframe (top) and ball and stick models (Bottom) of PLX3397 (blue) interacting with the bis-succinyl cyclodextrin ring

##
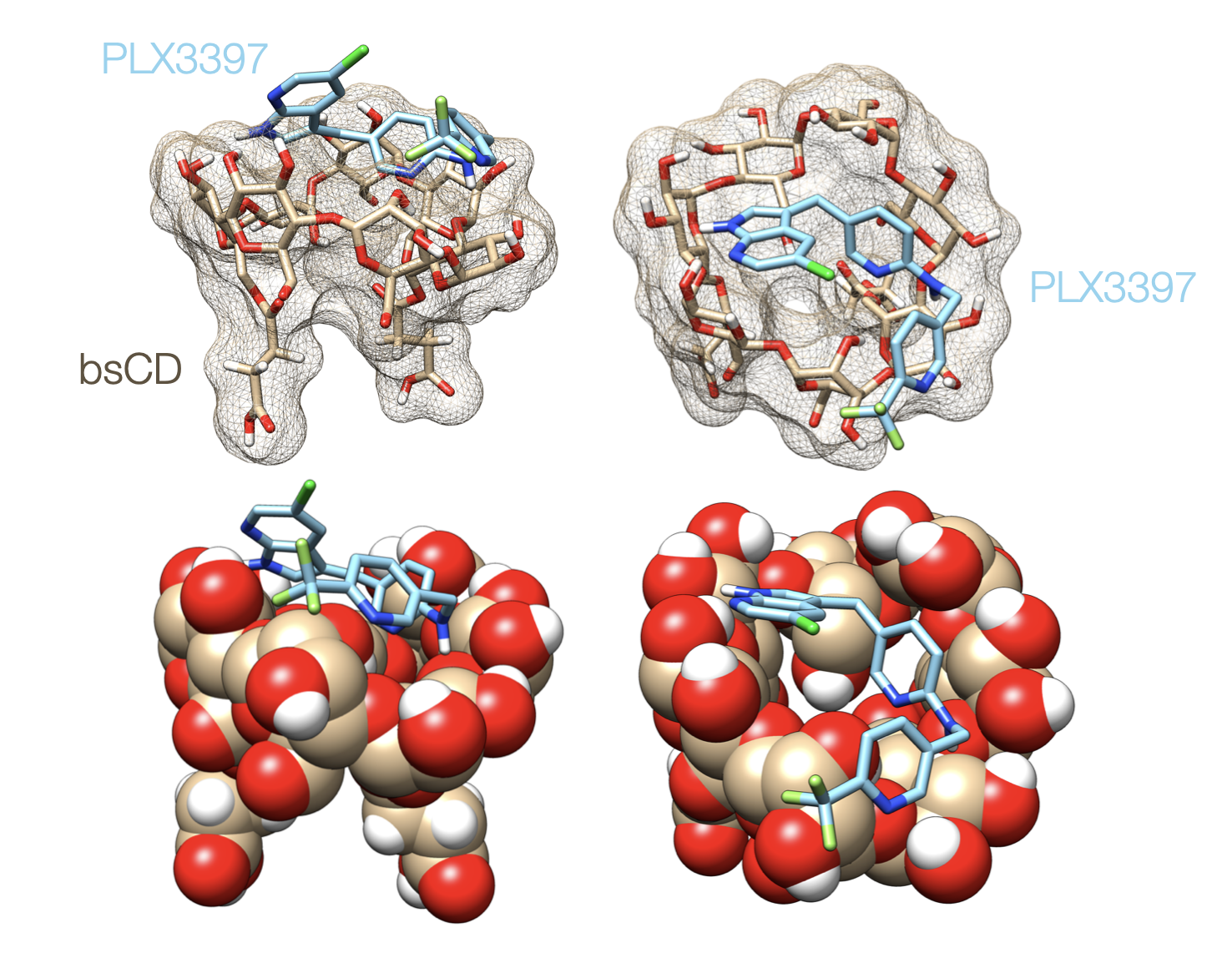


## Fig. S6: Histology of representative different treatment groups. The top panels show HE staining, and the bottom panels matching Spp1 immunohistochemistry (Spp1 red, DAPI blue; scale bar: 100 µm). Note the higher Spp1 levels in myeloid cells in tumors without treatment. Within 3 days of treatment with CANDI460, tumors start to involute, and Spp1 staining is less pronounced. Twenty days after treatment, the tumor is completely gone, showing largely subcutaneous fat cells without myeloid Spp1 cells.


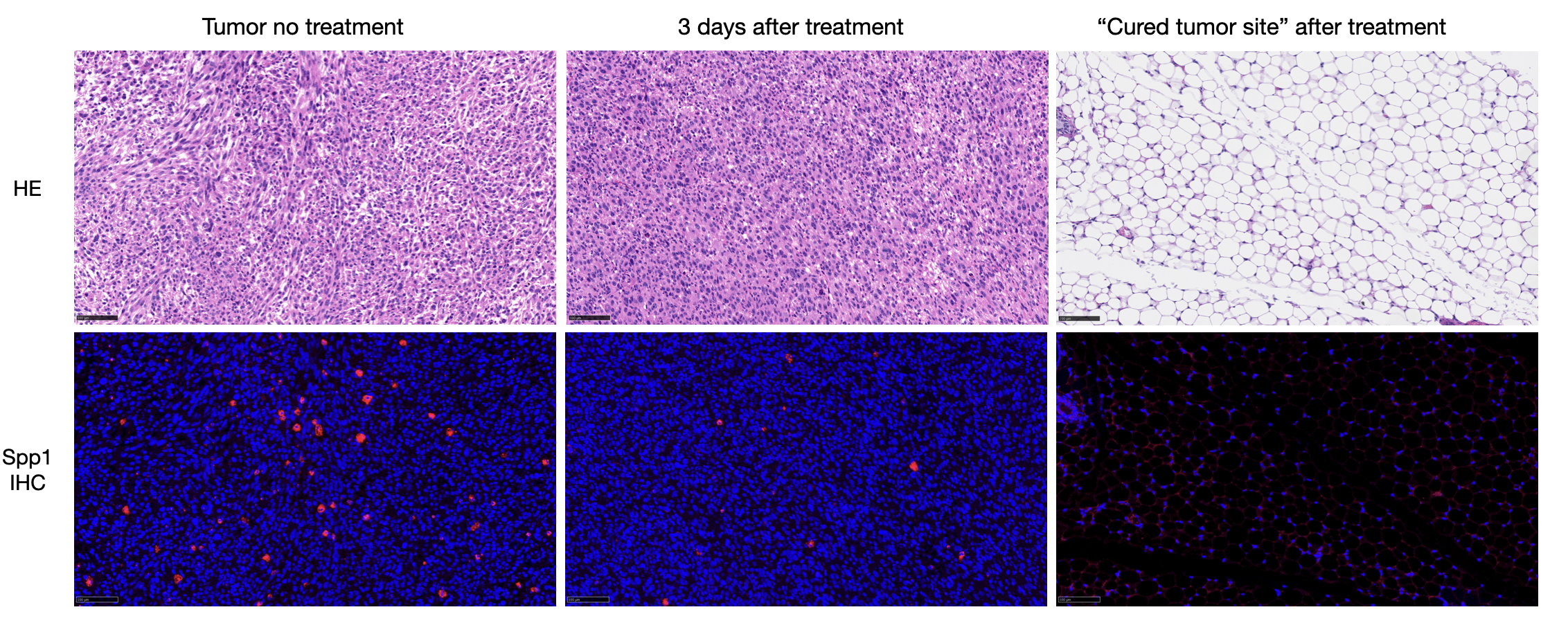


## Fig. S7: Flow cytometry. MC38 tumor-bearing mice were treated with CANDI460 or empty CANDI as control) for a single dose. Three days later, tumors were harvested and cells processed for flow cytometry. A. CD8 are highly elevated in CANDI460 treated animals. B. Macrophages and M2 markers were lower with CANDI460 treatment. (***: p < 0.001; **: p < 0.01; *: p < 0.05)


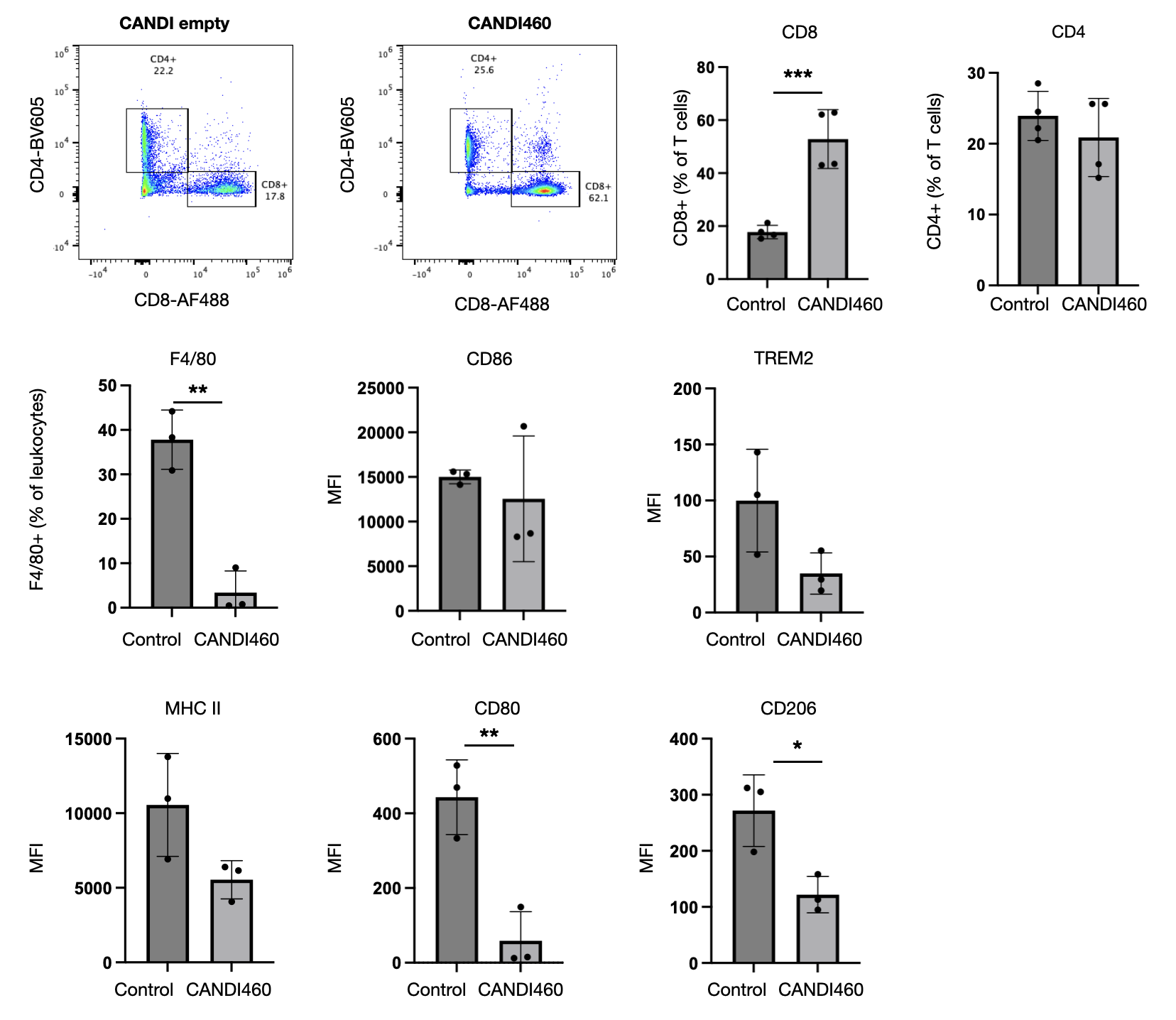


## Fig. S8: Detailed RNAseq analysis. Annotated volcano plot of bulk RNAseq of BMDM treated with CANDI460 or CANDI empty. Upregulated genes are shown on the right, and down-regulated genes with CANDi460 treatment are shown on the left. Key down-regulated myeloid genes include Spp1, Trem2, and Mrc1.
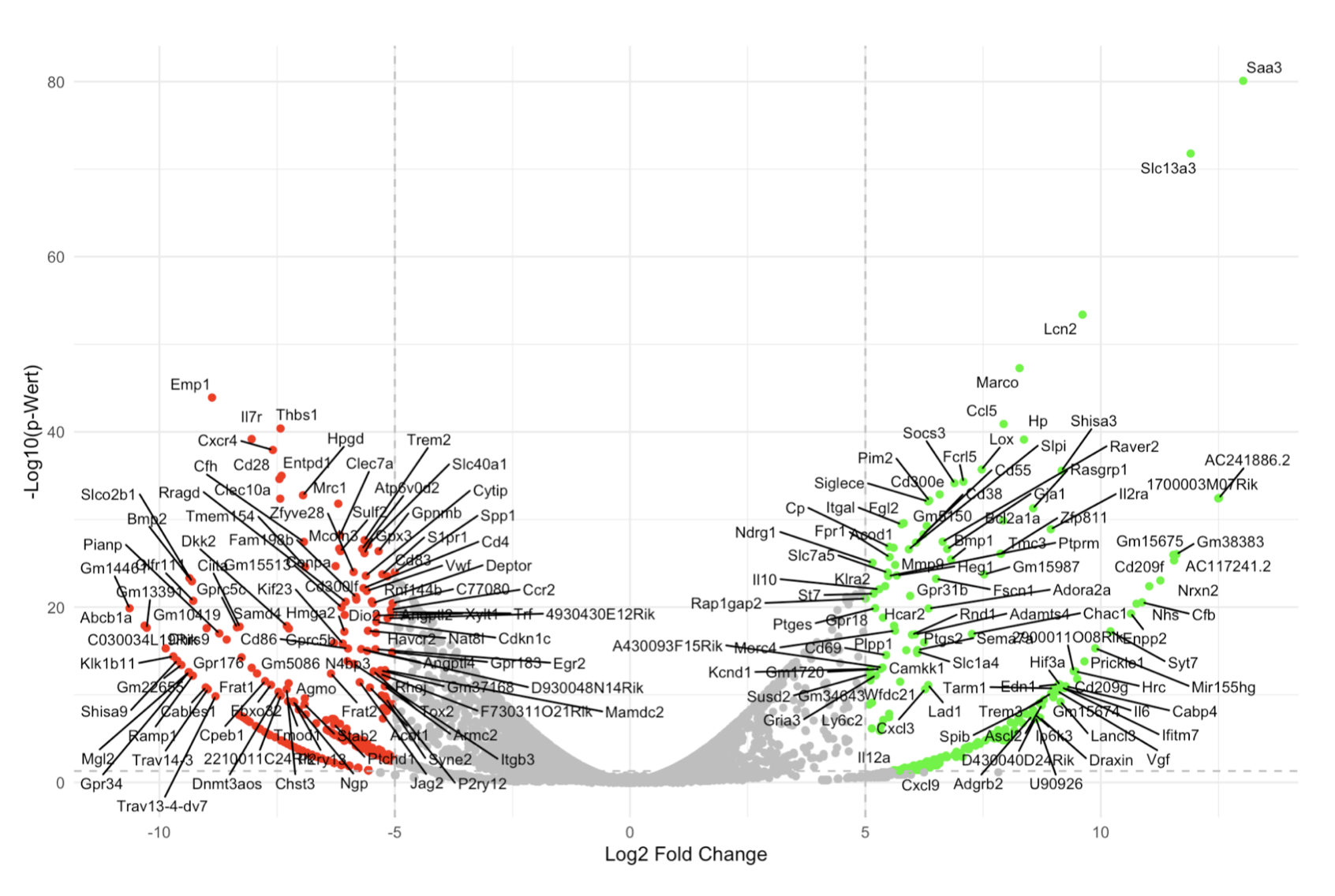


## Fig. S9. Cellular uptake of CANDI. A. Flow cytometry analysis of immune cell subsets in MC38 tumors 24 hours after intravenous injection of CANDI-AF647. Analysis shows a unique uptake of CANDI into myeloid cell populations (CD11b, CD11c/MHCII) but not into lymphoid (CD19, CD8, CD4) or other cell populations (MC38, NK1.1). Nearly 60% of all TAM contain CANDI-AF647. B. Uptake of CANDI-AF647 (white) into Spp1 BMDM (red; Nuclei stained with DAPI, blue). Note the high cellular uptake, best appreciated at higher magnification.
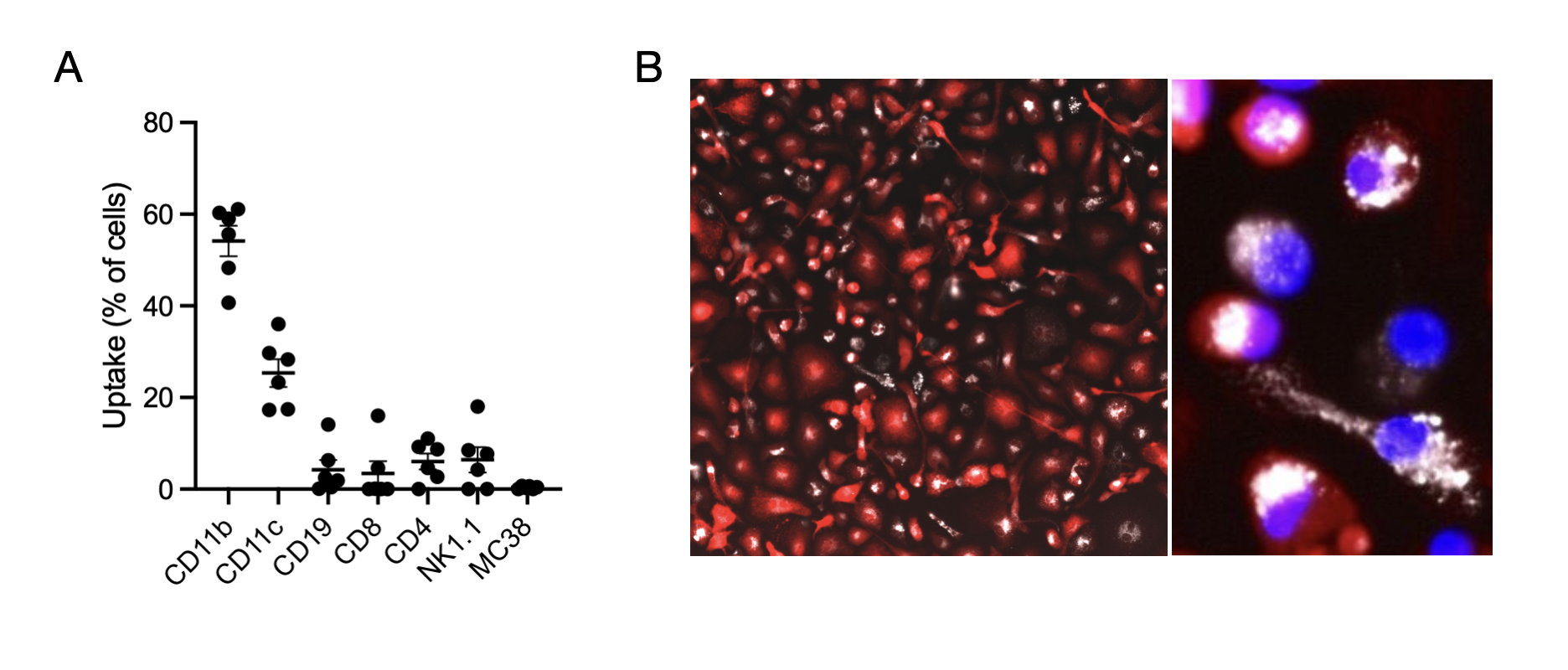


## Fig. S10: Efficacy determined by serial intravital imaging. Dorsal window chamber model of MC38-H2BGFP tumors implanted in SPP1-tdTomato mice. Tumors were imaged 8 days after implantation when the MC38 tumors had grown into solid masses (marked as Day 0, pre). Mice received two IV doses of CANDI460 as indicated. Note the gradual disappearance of the MC38-H2BGFP tumors. Five days after the last dose of CANDI, the tumors had completely disappeared. Scale bar: 500 µm.


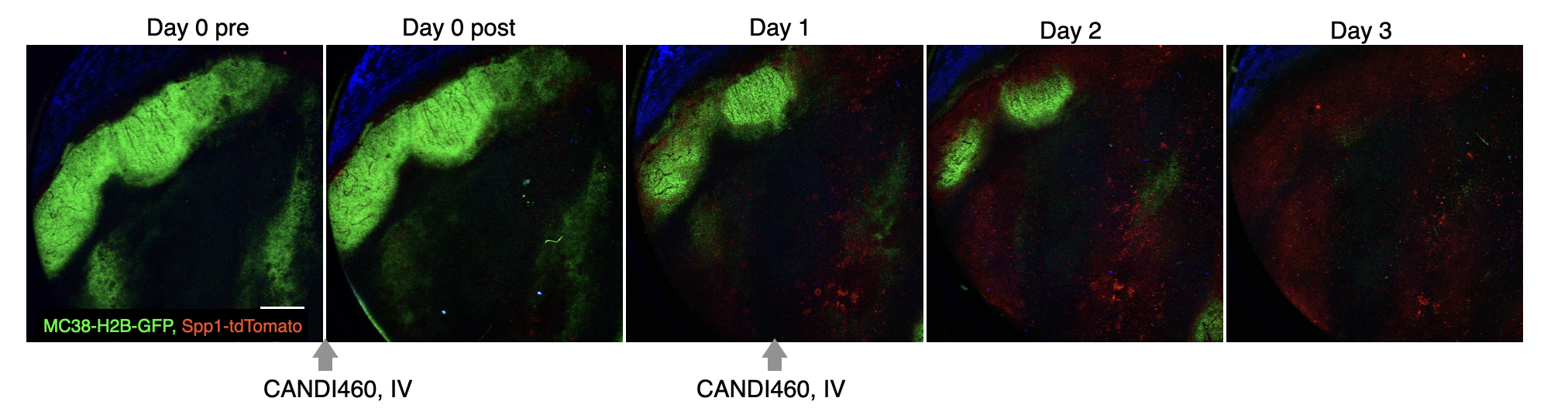


## Fig. S11: Intravital imaging to characterize extensive SPP1 cells. Dorsal window chamber model of MC38-TagBFP2 tumors implanted in crossed MerTK-GFP/Spp1-tdTomato mouse. A. Tumors were imaged 8 days after implantation when the MC38 tumors had grown into a solid mass. In this model, intratumoral TAMs appear MerTK positive (green). Most green cells are also positive for Spp1, but additional Spp1 cells are MerTK negative (see merge). B. 48.1 % of all TAM are Spp1 positive in this model. Scale bar: 50 µm.

##
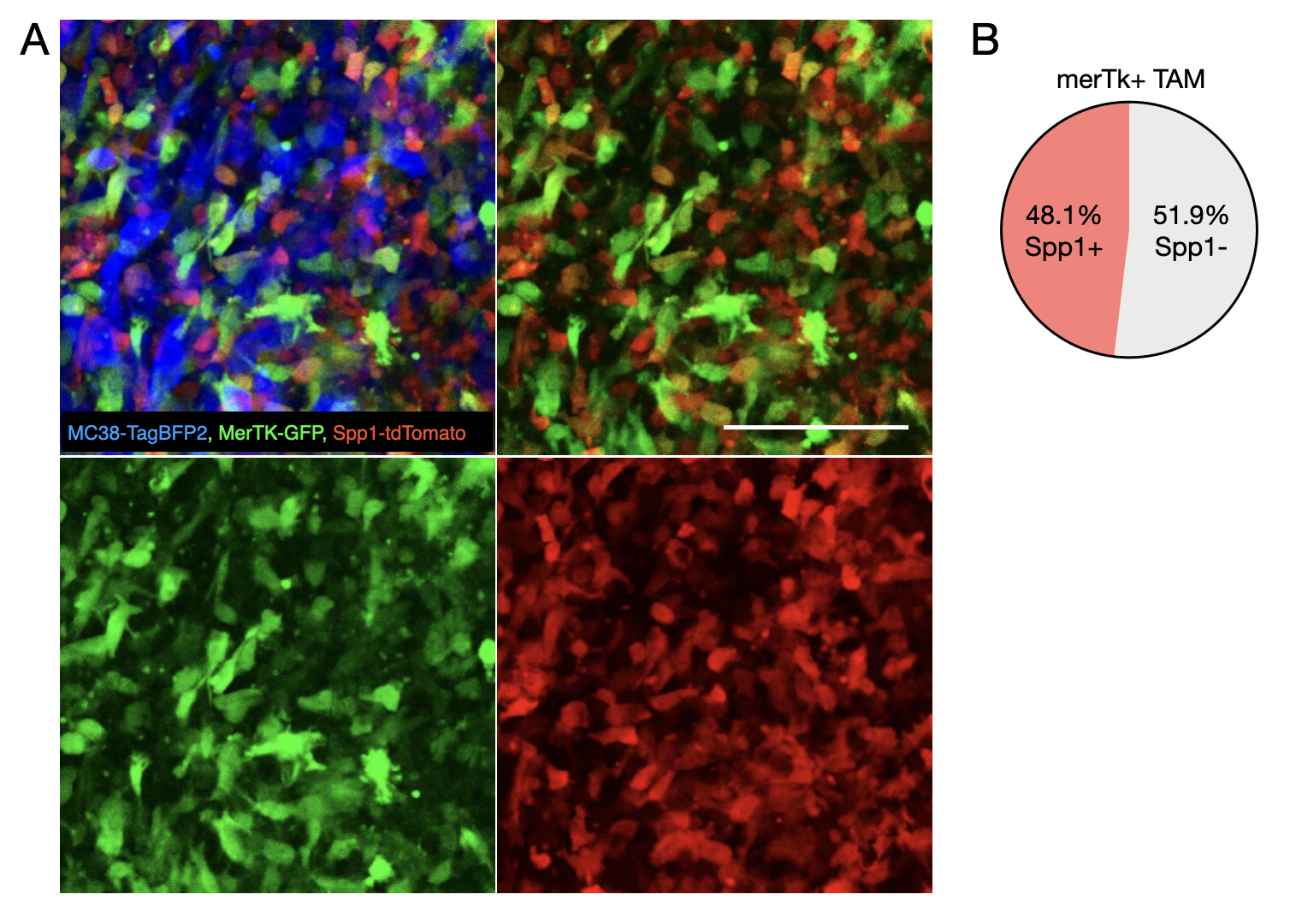


## Fig. S12: Efficacy in the B16 model. A. B16F10 tumors were implanted into the flank of mice on day 0. Tumor sizes were monitored over time by caliper measurements. B. Tumor volume changes plotted as a function of time with saline control (black) C. Same as in C but with three IV doses of CANDI460 administered on days 8, 10, and 12 (2 mg/mouse). D. aPD1 efficacy in the same model. E. Combination of CANDI460 and aPD1. Note the synergism. F. Waterfall plots of tumor changes in the four treatment groups at day 12. G. Kaplan Meier curve for different treatment groups (n=22).


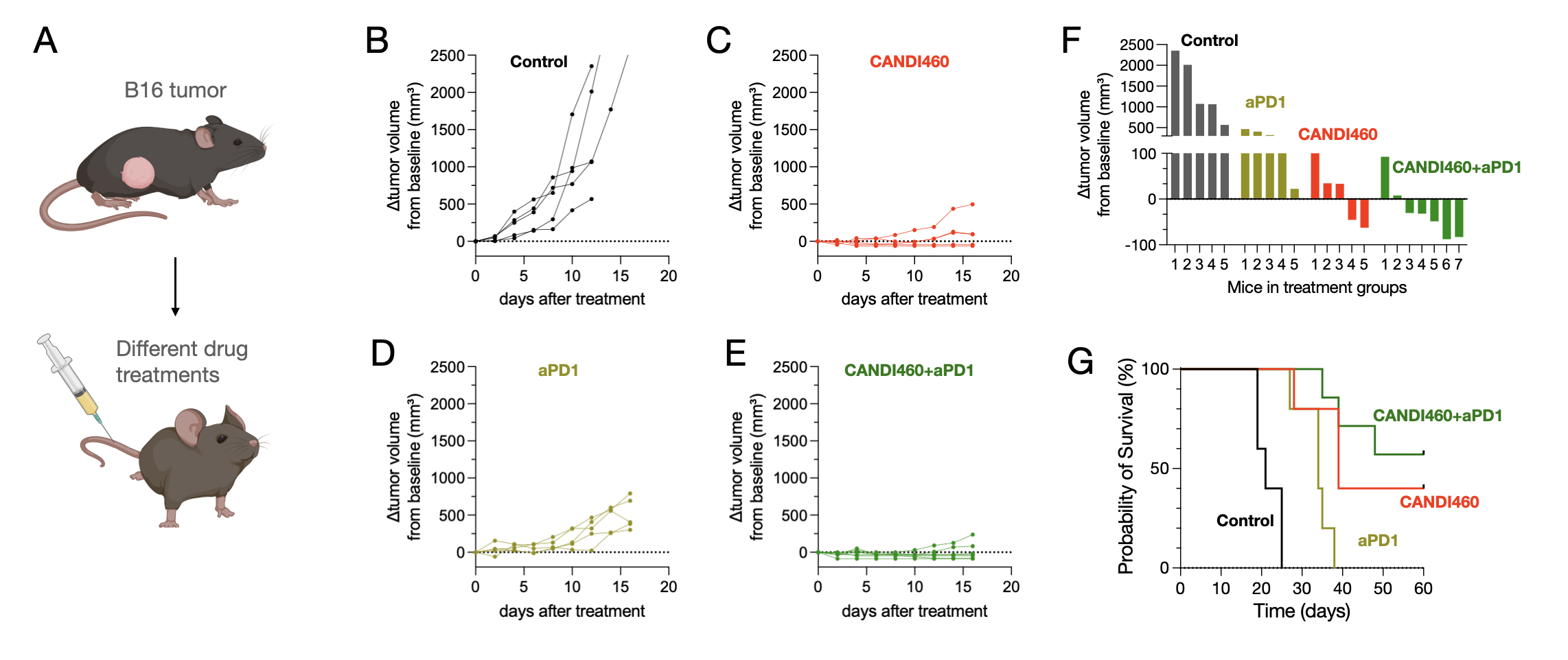


## Tables

**Table S1.** Mouse strains and experimental numbers

| Name | Manufacturer | Catalog Number | Application | N |
| --- | --- | --- | --- | --- |
| C57BL/6J-Spp1em1Msasn/J | Jackson Laboratory | Strain #:033731  RRID:IMSR_JAX:033731 | Donor mice for screens | 15 |
|  |  |  | IVM | 7 |
| MerTK-GFP | MGH colony | NA | IVM | 4 |
| C57BL/6J | Jackson Laboratory | Stock# 000664 RRID:IMSR_JAX:000664 | Tumor efficacy | 46 |
|  |  |  | RNA sequencing | 2 |
|  |  |  | Histology | 12 |
|  |  |  | Flow cytometry | 10 |
| Total (M≈F) |  |  |  | 96 |

**Table S2.** Overview of CANDI materials used in the study.

| Code | Payload | Drug loading | Comment |
| --- | --- | --- | --- |
| CANDI-111 | None | NA | Empty Nanoparticle (CANDI empty; CANDIE) |
| CANDI-114 | AF647 (covalent linkage) | 5 Fluorochromes/NP | Fluorescent version for IVM |
| CANDI-460 | R848 + PLX3397 | 0.0784 mg/mg | Drug loaded CANDI nanoparticle |

**Table S3.** List of antibodies and stains used.

| Antibody | Application | Target population | Source | Dye | Catalog # | Clone |
| --- | --- | --- | --- | --- | --- | --- |
| CD45 | Flow | Immune cells | BioLegend | AF700 | 103128 | 30-F11 |
| CD4 | Flow | CD4 T Cells | BioLegend | BV605 | 100548 | RM4-5 |
| CD8a | Flow | CD8 T Cells | BioLegend | AF488 | 100723 | 53-6.7 |
| F4/80 | Flow | Macrophage | BioLegend | BV711 | 123147 | BM8 |
| CD11c | Flow | DC | Invitrogen | PE-eF610 | 61-0114-80 | N418 |
| MHC-II | Flow | Antigen-presenting cell | Invitrogen | APC-eF780 | 47-5321-80 | M5/114.15.2 |
| CD11b | Flow | Myeloid cells | BioLegend | BV784 | 101243 | M1/70 |
| CD80 | Flow | Macrophage | Biolegend | BV421 | 104726 | 16-10A1 |
| CD206 | Flow | Macrophage | Biolegend | APC | 141707 | C068C2 |
| Live/Dead Aqua Amine | Flow | Live Dead Marker | Thermo |  | L34965 |  |
| CD86 | Flow | Macrophage | Biolegend | PE-Cy7 | 105013 | GL-1 |
| Fc block | Flow |  | BioLegend |  | 101320 | 93 |
| TREM2 | Flow | Macrophage | BioLegend | PE | 824805 | 6000000000 |
| PD-1 | Flow | T cells | BioLegend | PE | 114118 | RPM1-14 |
| CD90 | Flow | T cells | BD Bioscience | BV711 | 740647 | 53-2.1 |
| SPP1 | IHC | SPP1 positive cells | Abcam |  | ab218237 | EPR21138 |
| goat anti-rabbit IgG | IHC | SPP1 positive cells | Vector Labs | Biotin | BA-1000 |  |
| SA -DyLight 594 | IHC | SPP1 positive cells | Vector Labs | DL594 | SA-5594 |  |
